# Design and protocol for a pragmatic randomised study to optimise screening, prevention and care for tuberculosis and HIV in Malawi (PROSPECT Study)

**DOI:** 10.12688/wellcomeopenres.14598.3

**Published:** 2018-11-21

**Authors:** Peter MacPherson, Emily L Webb, David G. Lalloo, Marriott Nliwasa, Hendramoorthy Maheswaran, Elizabeth Joekes, Dama Phiri, Bertie Squire, Madhukar Pai, Elizabeth L Corbett

**Affiliations:** 1 Department of Clinical Sciences, Liverpool School of Tropical Medicine, Liverpool, UK; 2 Malawi-Liverpool-Wellcome Trust Clinical Research Programme, Blantyre , Malawi; 3 London School of Hygiene & Tropical Medicine, London, UK; 4 Helse Nord TB Programme, Department of Microbiology, College of Medicine, University of Malawi, Blantyre, Malawi; 5 Department of Public Health and Policy, University of Liverpool, Liverpool, UK; 6 Radiology Department, Queen Elizabeth Central Hospital, Blantyre, Malawi; 7 McGill International TB Centre, McGill University, Montreal , Canada

**Keywords:** Tuberculosis, HIV, public health, screening, diagnostics, treatment, randomised controlled trials, sub-Saharan Africa

## Abstract

**Background:** Adults seeking diagnosis and treatment for tuberculosis (TB) and HIV in low-resource settings face considerable barriers and have high pre-treatment mortality. Efforts to improve access to prompt TB treatment have been hampered by limitations in TB diagnostics, with considerable uncertainty about how available and new tests can best be implemented.

**Design and methods: **The PROSPECT Study is an open, three-arm pragmatic randomised study that will investigate the effectiveness and cost-effectiveness of optimised HIV and TB diagnosis and linkage to care interventions in reducing time to TB diagnosis and prevalence of undiagnosed TB and HIV in primary care in Blantyre, Malawi. Participants (≥ 18 years) attending a primary care clinic with TB symptoms (cough of any duration) will be randomly allocated to one of three groups: (i) standard of care; (ii) optimised HIV diagnosis and linkage; or (iii) optimised HIV and TB diagnosis and linkage. We will test two hypotheses: firstly, whether prompt linkage to HIV care should be prioritised for adults with TB symptoms; and secondly, whether an optimised TB triage testing algorithm comprised of digital chest x-ray evaluated by computer-aided diagnosis software and sputum GeneXpert MTB/Rif can outperform clinician-directed TB screening. The primary trial outcome will be time to TB treatment initiation by day 56, and secondary outcomes will include prevalence of undiagnosed TB and HIV, mortality, quality of life, and cost-effectiveness.

**Conclusions:** The PROSPECT Study will provide urgently-needed evidence under “real-life” conditions to inform clinicians and policy makers on how best to improve TB/HIV diagnosis and treatment in Africa.

**Clinical trial registration:** NCT03519425 (08/05/2018)

## Introduction

Tuberculosis (TB) is now the leading infectious cause of death worldwide ^1^. In 2016, there were an estimated 1.4 million deaths attributed to tuberculosis global, with an additional 0.4 million deaths from TB among people living with Human Immunodeficiency Virus (HIV) infection ^1, 2^. The countries of sub-Saharan Africa have been disproportionately affected by the HIV-TB co-epidemics. Following extremely rapid increases in TB incidence, prevalence and deaths during the 1990s and 2000s in the region that occurred concurrently with rapid increases in population HIV prevalence ^3^, TB rates have only begun to decline in the region in recent years ^1^. Although the expansion of coverage of effective antiretroviral therapy (ART) for treatment of HIV in many sub-Saharan countries has likely contributed to recent reductions in mortality, the pace of decline is unacceptably slow.

New impetus has been given to efforts to improve tuberculosis control by the recent-agreed global End-TB Strategy ^4^. This strategy, which was endorsed by WHO in 2015, demands global action and intensified research to address HIV-associated TB in 30-high HIV/TB burden countries that together comprise 87% of the global burden of TB ^2^. Key targets for the End-TB strategy include achievement by 2035 of a 90% reduction in TB incidence and a 95% reduction in TB mortality compared to 2015 ^4^.

Modelling studies have shown however that the End-TB targets will not be met without a step-change in efforts to improve the early diagnosis and effective treatment of all individuals with TB ^5^. Of concern remains low population TB case detection rates, and high case-fatality ratios, particularly among people living with HIV ^6^.

Adults seeking care at health facilities in sub-Saharan Africa are an important group to address in TB care and prevention programmes, as they have high prevalence of undiagnosed TB ^7^, a substantial burden of undiagnosed and untreated HIV ^8^, and high mortality rates if not promptly diagnosed and linked to treatment ^9^.

Our previous studies - similar to research from other countries in sub-Saharan Africa - have shown that the patient pathway from first health centre attendance, through diagnosis to successful treatment outcome is tortuous, with high rates of drop-out from care ^8, 10– 12^. Importantly, as well as having high mortality rates, individuals with symptoms of pulmonary TB who are not rapidly diagnosed may continue to transmit TB to others in the community, further limiting control efforts.

Although WHO guidelines promote intensified case-finding for TB among adults attending health facilities in high TB-prevalence settings ^13^, implementation of routine screening for TB is known to be suboptimal in many settings. Exit interviews done with patients leaving health facilities have shown that clinicians rarely conduct an initial symptom screen ^14, 15^. Moreover, even when symptoms of presumptive TB are reported, only a small fraction receive appropriate investigations for TB ^14, 15^. Thus, even at the earliest step of the TB diagnostic and care pathway, there are high rates of loss from the cascade.

In addition to low rates of TB screening, we have previously shown that only a small proportion (13%) of adults attending health facilities receive HIV testing, despite WHO and Malawi guidelines recommending a strategy of universal provider-initiated HIV testing and counselling (HTC) for all individuals attending health centres, regardless of reason ^8^. Uptake of HTC was highest among pregnant women attending for routine antenatal care, where considerable efforts have been undertaken to operationalise universal HTC as part of prevention of mother to child HIV transmission programmes. However, other groups, such as men and non-pregnant women have considerably lower rates of HIV testing completion ^8^; as they are attending with an acute care episode and have a higher prevalence of active tuberculosis, they may have substantially worse outcomes compared to pregnant women.

Through systematic reviews, meta-analysis, and prospective cohort studies we have shown that even if patients attending health centres are diagnosed with TB or HIV, they face considerable barriers to treatment initiation. In Africa, 18% of adults with sputum smear-positive tuberculosis will not initiate tuberculosis treatment promptly ^12^, whilst only a fifth of HIV-diagnosed adults will remain in care continuously to initiation of ART ^10^. Across both conditions a number of common factors hindering access to treatment have been identified, including: requirements to make multiple health centre visits for registration and assessment visits; debility; competing demands, including from work and education; and high out-of-pocket costs associated with visiting health centres ^16^.

When both HIV and TB are suspected or diagnosed, patients can face even greater challenges. Despite repeated calls for integration of HIV and TB care and prevention services, most clinic services remain vertically-organised. This means that patients often require multiple health centre visits on different days of the week to receive HIV and TB assessments and treatment, multiplying their adverse case-seeking costs and potentially worsening outcomes ^6, 16^.

We, and other, have therefore argued that new approaches are required to improve integration of HIV and TB screening, prevention and care services in health facilities in Africa that can provide same-day, same-clinic diagnosis and treatment linkage for both conditions at minimum inconvenience to patients ^16^. Such an approach, if effective, is likely to have large benefits for patients, public health, and for health systems by improving case detection and treatment access, reducing mortality, and mitigating the catastrophic costs associated with care-seeking. However, strong evidence for effectiveness obtained through robust randomised controlled trials is currently lacking.

Current TB screening approaches are reliant upon diagnostic tests with considerable limitations.

**Sputum smear microscopy** has been the mainstay of investigations for pulmonary tuberculosis for nearly a century. Although specificity is high, sensitivity remains unacceptably low, especially among HIV-positive adults ^17, 18^. Moreover, as sputum smears must be prepared, fixed and examined under light or fluorescence microscopy, infection control, quality control, throughput, and achievement of same-day diagnosis are challenging.

**TB culture** of sputum is slow (3–8 weeks, even with automated liquid culture systems), and relies upon availability of a high-quality laboratory. These requirements mean that whilst culture has importance for individual management of complex cases and for monitoring and evaluation, it is not practical as a point of care test ^19^.

**GeneXpert MTB/Rif** is an integrated and automated cartridge-based nucleic acid amplification test that can provide results for the detection of *M tuberculosis* and associated rifampicin resistance within two hours ^20^. There are two components to the test: the cartridge in which the biological sample is added to the assay, and a standalone unit in which cartridge is placed and where the nucleic acid amplification and detection takes place. The sensitivity of the Xpert assay is substantially higher than sputum smear microscopy ^20^, and the newest version (Xpert Ultra) shows pooled sensitivity (among HIV-positive and HIV negative samples) that is 5% higher than the first-generation assay, with a 12% gain among HIV-positive adults. WHO has endorsed GeneXpert MTB/Rif as the first line test among adults suspected to have multidrug resistant TB or HIV-associated TB.

Despite these advantages, there are some barriers to the widespread implementation of Xpert in low-resource, high TB prevalence settings. In particular, even at current concessional pricing for low-resource settings, Xpert is prohibitively expensive as a first line test for most national programmes.

**Chest radiography** has high sensitivity for pulmonary TB even in HIV coinfection ^21, 22^, and continues to play an important role in TB diagnosis in high-income settings. Although chest x-ray has been used for many years as a diagnostic tool (usually at the end of screening algorithms), widespread implementation in high prevalence settings has been limited by poor access to high quality equipment and expert radiologists, low specificity (leading to over-diagnosis of TB if chest x-ray alone is used) and high inter-reader variability ^22^. Recent advances in digital chest x-ray technologies have reinvigorated interest in the use of chest x-ray as an initial triage tool in primary care in Africa.

Chest X-ray may also be used as a triage test for TB ^22^. In this triage approach, individuals with any abnormality identified on chest x-ray undergo confirmatory microbiological testing. Using a point-of-care high specificity molecular sputum testing for confirmatory testing (e.g. Xpert MTB/Rif) could allow accurate same-day TB diagnosis and treatment initiation in primary care.

In December 2016, WHO released a new evidence review and guidance ^22^ for chest x-ray TB triage that used data from systematic review to model the potential effectiveness of TB screening algorithms, and showed that triage using chest x-ray, followed by GeneXpert MTB/Rif could substantially outperform other approaches.

Currently, countries such as Malawi have low coverage of radiology services, including trained radiologists. Computer-assisted detection (CAD) software - statistical algorithms used to classify digital images - is now available, and can be integrated within new digital x-ray units to provide immediate triage ^23^. WHO recently systematically reviewed available evidence for one CAD system (CAD4TB, Delft Imaging Systems, Netherlands). Across 13 studies conducted in a variety of populations, sensitivity was as high as reading by radiologists, although specificity was lower necessitating microbiological confirmation of TB ^22^. Whilst promising, WHO recommends that *“CAD can be used for TB detection for research, ideally following a protocol that contributes to the required evidence base for guideline development” ^22^*.

In summary, adults with symptoms of tuberculosis in Malawi face considerable health systems delays, large out-of-pocket expenses, and have a high risk of mortality before diagnosis and treatment. To achieve the End-TB Strategy goals, a package of same-day, same-clinic diagnosis and treatment linkage interventions for both TB and HIV are urgently required. In an individually-randomised, open, three-arm controlled trial, The PROSPECT Study will investigate whether optimised TB and HIV diagnosis and treatment linkage interventions are cost-effective in reducing time to TB treatment initiation, and in improving case detection.

## Methods

### Study design

The PROPSECT Study is an open, three-arm pragmatic randomised controlled trial.

### Study hypothesis

The PROSPECT Study will test the hypothesis that an optimised same-day TB/HIV screening and treatment linkage intervention for adults with presumptive tuberculosis in primary care could result in important improvements in eight-week case detection, treatment initiation and mortality.

### Objectives

I. Among adults with TB symptoms attending primary care in Malawi, to investigate the effectiveness of an optimised same-day screening algorithm consisting of rapid HIV testing, computer-assisted CAD4TB chest x-ray triage and, if abnormal, Xpert MTB/Rif rapid sputum molecular testing, and linkage to treatment.
II. In a nested diagnostic accuracy study evaluate the sensitivity and specificity of computer-assisted chest x-ray triage compared to classification by radiologists and bacteriological diagnosis.
III. Undertake a cost-utility analysis of the PROSPECT interventions to estimate the incremental cost per QALY gained from providing optimised TB and HIV diagnosis and linkage to care.

### Study site and population

The study will be done at Bangwe Health Centre, a busy health centre located in a densely populated urban neighbourhood of Blantyre, Malawi. Adult prevalence of tuberculosis and HIV in Blantyre are >900 per 100,000 ^24^ and 18.5% respectively ^25^.

TB diagnostics available at the study clinic include sputum smear fluorescence microscopy with LED microscopes, and Xpert MTB/Rif. Although Malawi and World Health Organization (WHO) guidelines recommend screening for tuberculosis for all individuals attending health facilities with acute illness (with Xpert preferred as the first-line investigation for symptomatic HIV-positive individuals), our previous research shows that TB diagnostics are underutilised ^26^.

At the study clinic, comprehensive HIV care is available and includes: routine provider-initiated HIV testing and counselling, screening and treatment of opportunistic infections, provision of chemoprophylaxis, and treatment with antiretroviral therapy. Malawi National Guidelines have recommended a “test and treat” approach to HIV since 2015, with all individuals diagnosed with HIV being eligible for antiretroviral therapy. Our previous research has shown however that coverage of HIV testing and rates of linkage to antiretroviral therapy are suboptimal ^8, 10^, as in many other African settings.

Study participants will be adults aged 18 years or older who attend Bangwe Health Centre for acute care with symptoms of tuberculosis (cough of any duration). As this is a pragmatic randomised trial that aims to provide evidence for policymakers under “real-life“ conditions, eligibility criteria will be broad, and will reflect the characteristics of adults attending primary health centres in Southern Africa to maximise generalisability. We will exclude: individuals who are taking treatment for tuberculosis, or who have taken tuberculosis treatment in the preceding 6-months; individuals taking isoniazid preventive therapy; and individuals who live outside of Blantyre or plan to relocate outside of Blantyre in the next six months.

Research assistants based at the clinic registration desk will screen all daily attenders against eligibility criteria. As individuals may attend the clinic on more than one occasion during the study period, the research assistant will record a digital fingerprint (Simprints, Cambridge, UK) from all clinic attenders to ensure that repeat clinic attendance episodes are recorded and so removing the potential for duplication in trial recruitment. Where the number of eligible clinic attenders exceeds recruitment capacity, we will recruit participants up to a daily limit, with details finalized pending completion of pilot work showing the number of eligible participants per day.”

### Randomisation and blinding

Participants will be individually randomly allocated in a 1:1:1 ratio into one of three groups.

Enrolment and baseline questionnaires will precede randomization. Randomisation will be done by research assistants using a random number allocation schedule running on study data-collection electronic tablets. Because of the nature of the study and the interventions offered, it will not be possible to blind participants or research assistants to allocation groups. Nevertheless, extensive steps will be taken to ensure that research assistants undertaking outcome assessments are blinded to participants’ group. Additionally, the investigators, including the chief investigator and trial statistician, will remain blinded to allocation groups until final analysis. No unblinded interim analysis will be conducted.

### Interventions

*All participants*

All participants will complete a baseline questionnaire ( Supplementary File 1), that will record demographic and clinical characteristics (including previous HIV and TB care), as well as geolocation information ^16^ to facilitate home tracing if participants don’t attend for outcomes assessments. All participants will additionally complete the EuroQoL EQ5D (Chichewa) tool (English version validated for the UK available here) to measure health-related quality of life.

*Group 1: Standard of care*

**Figure 1.**
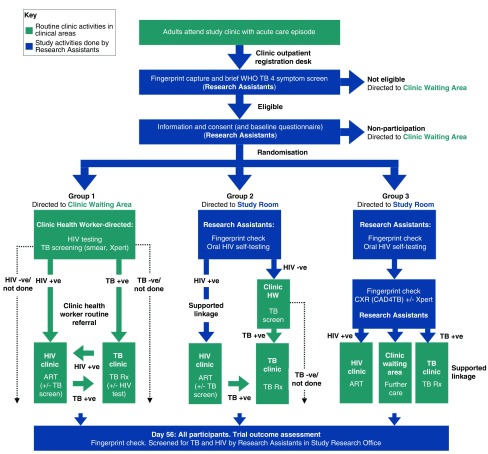
PROSPECT Trial Design. WHO: World Health Organization, TB: tuberculosis, HIV: Human immunodeficiency virus, +ve: positive, -ve: negative, ART: antiretroviral therapy, Rx: treatment, +/- : with, or without.

Interventions available to participants allocated to Group 1 are intended to mirror the current standard of care for HIV and TB screening and linkage to care under routine conditions in primary care in Malawi ( Figure 1). This will ensure that the incremental cost-effectiveness of interventions offered in Groups 2 and 3 can be compared to a standard care that reflects “real-life” practice.

Participants allocated to Group 1 will be directed to the routine facility waiting area to be seen by facility health workers. Screening for HIV and tuberculosis will be directed by the routine facility health workers in accordance with Malawi National Guidelines, and without any further trial interventions.

*Group 2: Optimised HIV screening and linkage to care*

Participants allocated to Group 2 will be offered a supervised HIV self-testing intervention using oral fluid rapid diagnostic kits (OraQuick ^®^ HIV-1/2 rapid antibody test kits, manufactured in Thailand for: OraSure Technologies, Inc. Bethlehem, PA, USA). Participants will have their identity validated by fingerprint scanning, will be provided with brief pre-test counselling and instruction and demonstration in the use of the oral fluid kits by research assistants, and will be asked to self-test in a private clinic area, with support from the research assistant.

Participants who test HIV-negative will be referred to the clinic waiting area with a copy of their HIV test result, and will be assessed by the routine facility health workers as in Group 1.

Participants whose oral fluid test is reactive will have confirmatory HIV testing performed by trained research assistants using rapid diagnostic kits and following a testing algorithm as recommended by WHO and Malawi National Guidelines.

Participants with confirmed HIV infection will be supported to register for HIV care at the HIV care clinic located within the study health centre and will be provided with a written appointment date to attend for initial antiretroviral therapy assessment and initiation appointment. As part of HIV treatment assessments, Malawi National guidelines recommend screening for active tuberculosis. In this group, participants’ TB screening will be directed by facility HIV clinic health workers - not by the study team - with sputum smear microscopy and GeneXpert MTB/Rif available to clinicians through routine clinic services, and without further study intervention.

*Group 3: Optimised TB and HIV screening and linkage to care*

Participants allocated to Group 3 will be directed to a research study room located in a separate area of the clinic. Prior to intervention delivery, a digital fingerprint check will be repeated to validate participants’ identity and minimise any potential for contamination between groups. Participants will be offered supervised HIV self-testing, with confirmatory testing and post-test counselling as in Group 2 above.

All participants allocated to Group 3 will additionally be offered a digital chest x-ray (MinXray Inc., USA) that will be pre-processed and quality-checked by a study radiographer. Digital chest x-ray images will then be immediately evaluated by the CAD4TB computer-aided TB triage algorithm (Delpht Imaging Systems, Netherlands). Application of the CAD4TB algorithm will provide a TB score ranging from 0 to 100. Based on a pilot evaluation of chest x-rays taken from adults attending the study clinic with cough and analysed by Delpht Imaging services using CAD4TB version 4.12.2, we set a threshold CAD4TB score of 45. Participants with a CAD4TB score at or above this threshold will be classified as having “high probability of tuberculosis”, whilst those below this threshold will be classified as having “low probability of TB”.

Participants whose chest x-rays are classified as having “high probability of TB” will be invited to submit a single sputum sample (induced with saline nebulization if necessary) for testing by GeneXpert MTB/Rif (Cephaid, USA) in the study clinic. Participants whose GeneXpert MTB/Rif results demonstrate the presence of *M. tuberculosis* will be supported to register for tuberculosis treatment on the same day at the TB clinic within the study health facility.

Participants whose chest x-ray are classified as being “low probability of TB” and those whose GeneXpert MTB/Rif tests are negative will be referred to the clinic waiting room to be seen by facility health workers, with a written report of the results of the investigations they have completed.

### Definitions

**Microbiologically-confirmed tuberculosis** will be defined by: A participant with: a documented positive GeneXpert MTB/Rif result for *Mycobacterium tuberculosis* on at least one sample of sputum taken for study or routine clinical purposes; or documented growth of *Mycobacterium tuberculosis* and positive speciation using MPT 64 antigen tests on at least one culture of sputum taken for study or routine clinical purposes; or documented identification of acid fast bacilli on at least one sputum sample taken for study or routine clinical purposes and examined by sputum smear microscopy.

**Clinically-diagnosed tuberculosis** will be defined by: A participant who does not fulfil the criteria for microbiologically-confirmed TB but has documented evidence of having been diagnosed with active TB by a clinician or other medical practitioner who has decided to give the patient a full course of TB treatment. This definition includes cases diagnosed on the basis of X-ray abnormalities or suggestive histology and extrapulmonary cases without laboratory confirmation. Clinically-diagnosed cases that are subsequently found to be bacteriologically-positive (before or after starting treatment) will be reclassified as bacteriologically-confirmed.

**Pulmonary tuberculosis (PTB)** will be defined by: A participant with bacteriologically-confirmed or clinically diagnosed case of TB involving the lung parenchyma or the tracheobronchial tree. Miliary TB will be classified as PTB because there are lesions in the lungs. Tuberculous intra-thoracic lymphadenopathy (mediastinal and/or hilar) or tuberculous pleural effusion, without radiographic abnormalities in the lungs, constitutes a case of extrapulmonary TB. A patient with both pulmonary and extrapulmonary TB will be classified as a case of PTB.

**Extrapulmonary tuberculosis (EPTB)** will be defined by: A participant with bacteriologically-confirmed or clinically-diagnosed case of TB involving organs other than the lungs, e.g. pleura, lymph nodes, abdomen, genitourinary tract, skin, joints and bones, meninges.

**Initiation of tuberculosis treatment** will be defined by: A participant in whom there is documented evidence of commencement of anti-tuberculosis treatment, either by: inspection of the participant-carried national tuberculosis treatment card; or inspection of the facility tuberculosis treatment register; or inspection of TB treatment medication bottles or pill boxes.

**Initiation of antiretroviral therapy** will be defined by: A participant in whom there is documented evidence of commencement of combination antiretroviral therapy treatment, either by: inspection of the participant-carried national HIV programme treatment card; or inspection of the facility antiretroviral therapy treatment register; or inspection of antiretroviral therapy medication bottles or pill boxes.

**Successful tuberculosis treatment outcome** will be defined by: A participant in whom tuberculosis treatment is initiated for bacteriologically-confirmed pulmonary tuberculosis, and who has documented evidence in their national tuberculosis treatment card of being cured of TB, being either sputum smear- or culture-negative in their last month of treatment and on at least one previous occasion; or a participant with documented evidence of having completed TB treatment without evidence of failure (that is sputum smear- or culture-positive at month 5 or later during treatment) but with no record to show that sputum smear or culture results in the last month of treatment and on at least one previous occasion were negative, either because tests were not done or because results are unavailable.

### Adverse events

As this is a pragmatic randomised trial and no new investigational products are being evaluated, we anticipate only a small number of adverse events. Nevertheless, we will ensure that case definitions, standardised operating procedures and a reporting protocol will be in place to record all adverse events.

The following adverse events will be systematically recorded and reported:

- Misclassification or misinterpretation of results leading to a participant starting TB therapy in error
- Misclassification or misinterpretation of results leading to a participant starting HIV treatment in error
- Breach of confidentiality following TB or HIV diagnosis
- Needlestick injuries

### Trial outcomes

The primary trial outcome will be time in days – from Day 0 up to but not including Day 56 – to tuberculosis treatment initiation, evaluated at Day 56 following randomization.

Analysis of the primary outcome will be done on an intention to treat basis, with all participants allocated to trial groups included and analysed in the group to which they were randomized (regardless of which intervention was received). We will make three pair-wise comparisons *(Group 2 vs. Group 1; Group 3 vs. Group 2; and Group 3 vs. Group 1)*.

This primary endpoint has been chosen because reducing time to initiation of treatment could have important individual and public health benefits. Assessment over eight weeks has been selected because: (i) TB culture is typically completed within 8 weeks, (ii) mortality is highest during this period ^27, 28^, and (iii) previous trials and our previous research show that TB treatment initiations plateau by 8 weeks ^27^.

The secondary trial outcomes will be:

- The proportion of randomised participants initiated onto tuberculosis treatment on the same day as randomisation, with the numerator being participants who were initiated on tuberculosis treatment on Day 0, and the denominator being all randomised participants.
- The proportion of randomised participants with undiagnosed/untreated microbiologically-confirmed pulmonary TB at Day 56, with the numerator being participants with microbiologically-confirmed tuberculosis (either sputum culture, or sputum Xpert, or sputum smear microscopy positive on a sample taken on Day 56) and who are confirmed not to be taking tuberculosis treatment on Day 56 (including participants who have previously initiated tuberculosis treatment, but have defaulted or stopped treatment – regardless of reason – for at least one week). The denominator will be all randomised participants.
- The proportion of randomised participants with undiagnosed/untreated HIV at Day 56, with the numerator being participants with positive confirmatory HIV test results at Day 56 and who are not taking antiretroviral therapy (regardless of previous HIV test results during or before the study period), and the denominator being all randomised participants.
- Time in days - from Day 0 up to but not including Day 56 - to initiation of antiretroviral therapy among participants with positive confirmatory HIV test results at Day 56 and who were not taking antiretroviral therapy at Day 0.
- The proportion of randomised participants reported to have died by Day 56, with the numerator being participants confirmed to have died through home tracing visits or TB treatment records, and the denominator being all randomised participants
- The proportion of TB cases with a successful TB treatment outcome. The numerator will be participants who were initiated onto tuberculosis treatment (either microbiologically-confirmed or clinically-diagnosed tuberculosis) up to, but not including Day 56, and who have a successful TB treatment outcome (either cured or completed treatment) at 6-months after starting treatment. The denominator will be all participants confirmed to have initiated tuberculosis treatment between Day 0 and up to, but not including Day 56.
- Mean difference in EuroQoL EQ5D utility score at Day 56, adjusting for participants’ EQ5D utility score measured at Day 0.
- Mean difference in EuroQoL EQ5D visual analogue scale score, adjusting for participants’ EQ5D visual analogue scale score measured at Day 0.
- Incremental cost-effectiveness per quality-adjusted life year gained

### Planned subgroup analyses

In pre-planned exploratory analysis, we will stratify analysis of the primary outcome by: sex (male vs. female); and microbiological status (bacteriologically-confirmed TB vs. clinically-diagnosed TB).

Additionally, we will undertake a Bayesian analysis of the primary trial outcome ^29^. Prior distributions for the proportion initiating TB treatment under each intervention strategy will be elicited from key stakeholder groups. We anticipate that key stakeholders will include: community members; clinic health workers; researchers; TB/HIV experts; and policymakers (Malawi, regional, and international). Before eliciting stakeholders’ prior beliefs for trial interventions, we will provide a series of “warm-up” vignettes based around familiar events such as the probability of a football team winning, or the probability of it raining tomorrow. Each stakeholder will then be asked to make ten guesses for the percentage of participants who will initiate TB treatment under each intervention strategy.

### Outcome evaluation

Following completion of trial interventions, all participants in each of the three groups will be given a written appointment card to attend a follow-up assessment at the study research clinic room 56 days after randomization (or as close as possible after this date). They will also be issued with a voucher that they can use to reimburse the cost of transport to the clinic for this assessment. Participants who don’t attend their day 56 appointment will be traced to home.

To evaluate the primary trial outcome (time to tuberculosis treatment initiation), Research Assistants will undertake a detailed questionnaire to record the date of TB treatment initiation, and verify by inspecting participant-carried TB treatment cards, medication and clinic TB treatment registers.

To evaluate the prevalence of undiagnosed tuberculosis, Research Assistants will collect sputa from all participants. Samples will be transported to the TB Research Laboratory at the College of Medicine of Malawi, where they will be cultured for tuberculosis using the MGIT system, undergo smear examination using fluorescence microscopy, and tested using the GeneXpert MTB/Rif assay. Positive TB results will be reported to participants within three days of receipt (including by home tracing), and participants will be supported to register for TB treatment at the health facility. Participants with Rifampicin resistance detected on GeneXpert will be traced and supported to access the TB clinic at the Queen Elizabeth Central Hospital for further clinical assessment and evaluation for treatment.

To evaluate the prevalence of undiagnosed HIV at Day 56, Research Assistants will offer all participants HIV testing, unless they are confirmed to be taking antiretroviral therapy. All participants requiring additional care will be supported to access either the HIV clinic, TB clinic, or outpatient clinic at the study clinic as required. Additionally, we will support referral and access to Queen Elizabeth Central Hospital should further specialist care be required.

To evaluate health-related quality of life (HRQoL), we will use the EQ-5D-3L. The EQ-5D is a generic HRQoL measure, and was translated into Chichewa following international and EuroQoL guidelines. The EQ-5D-3L tool will be administered to all trial participants at baseline, and on Day 56.

### Statistical methods

The primary trial outcome, time to TB treatment initiation, will be compared between pairs of groups among all randomised participants. To evaluate the relative effects of the HIV and TB screening/linkage interventions, we will make three pairwise comparison *(Group 2 vs. Group 1; Group 3 vs Group 2; and Group 3 vs Group 1)*. Our pilot data show that 17% of adults with TB symptoms will initiate TB treatment under routine screening conditions within 8-weeks.

Using formula for the proportional hazards model developed by Schoenfield ^30^, and inflating by 5% for loss to follow-up, a total sample size of 1455 participants (485 per group) gives at least 80% power to detect at least a cumulative hazard ratio for TB treatment initiation of 1.5 comparing Group 2 to Group 1, and a hazard ratio of 1.41 comparing Group 3 to Group 2, at 5% significance level. Additionally, under these assumptions, 485 participants per group would give 80% power to detect a hazard ratio of at least 1.50 comparing Group 3 to Group 1.

All statistical analysis will be conducted in accordance with a pre-published statistical analysis plan ( Supplementary File 2). Trial reporting will follow CONSORT Guidelines. We will report baseline characteristics of randomised participants, stratified by allocated group.

Analysis of the primary and secondary outcomes will be done on an intention to treat basis, with all participants allocated to trial groups included. We will index the day of recruitment to be Day 0 and outcome assessment will take place on, or as close to possible after, Day 56. Initiation of tuberculosis treatment will be defined by a participant in whom there is documented evidence of commencement of anti-tuberculosis treatment between Day 0 and up to, but not including Day 56. Time to TB treatment outcome analysis will be right-censored on day 56 if TB treatment is not initiated. We will estimate per-group median times to TB treatment initiation, and plot cumulative hazard function graphs.

To investigate the relative effectiveness of interventions on the cumulative hazard of TB treatment initiation, we will conduct log rank tests and construct Cox proportional hazard regression models to estimate hazard ratios and 95% confidence intervals for each pairwise comparison *(e.g. Group 2 vs. Group 1, Group 3 vs. Group 2, and Group 3 vs. Group 1)*. Log-log plots will be examined and Schoenfeld residuals used to test the proportional hazards assumption.

To analyse binary secondary outcomes (proportion with same-day tuberculosis treatment initiation, proportion with undiagnosed/untreated pulmonary tuberculosis, proportion with undiagnosed/untreated HIV, proportion reported to have died by Day 56, proportion with successful TB treatment outcome), we will construct log-binomial regression models to estimate relative risk ratios and 95% confidence intervals, comparing between pairs of groups. We will additionally compare between pairs of groups the time to antiretroviral therapy initiation among participants with previously untreated HIV using Cox regression models.

To evaluate the effect of interventions on health-related quality of life, we will use ANCOVA analysis to compare the mean EQ5D utility scores and visual analogue scale scores measured at Day 56 between pairs of groups, adjusting for participants’ values measured at Day 0.

For the preplanned subgroup analysis of the primary trial outcome we will construct Cox proportion hazard regression models including a term for either sex (male or female) or microbiological TB status (either microbiologically confirmed or clinically-diagnosed) to estimate hazard ratios and 95% confidence intervals. We will use the likelihood ratio test to look for interactions between sex/microbiological-confirmed TB and trial group.

### Bayesian analysis of primary trial outcome

Using within and between participant elicited probability distributions, we will construct stakeholder group-specific pooled prior probability distributions (known as a “community of priors”). Each prior will be converted to a log-hazard ratio scale and fitted to a normal distribution, allowing comparison between stakeholder groups of the similarity in support of opinions of effectiveness and of uncertainty.

Using Bayes’ theorem we will combine elicited stakeholder group-specific log hazard ratio prior distributions with log-likelihood hazard ratio distributions from each pairwise comparison being made in the PROSPECT Study to construct posterior probability distributions. All analysis will be done in R and posterior mean hazard ratios and 95% credible intervals will be estimated by taking draws from the posterior distributions using the No-U-Turn Sampler (NUTS) implemented with Stan.

### Additional nested analysis

WHO has recommended that *“Computer aided diagnosis can be used for TB detection for research, ideally following a protocol that contributes to the required evidence base for guideline development” ^22^*. The PROSPECT Study therefore offers opportunity to undertake a nested evaluation to contribute to the evidence base for the diagnostic accuracy (as well as effectiveness) of the CAD4TB platform.

Participants for this nested evaluation will be adults recruited to the main PROSPECT Study trial, and who complete a Day 56 outcome TB screening assessment. As part of this outcome assessment, all participants will undergo CAD4TB classification of digital chest x-ray, as well as sputum testing by GeneXpert, and TB liquid automated culture. All digital chest x-rays taken from participants at outcome assessment will be uploaded to the password protected and secure MinXray online picture archiving and communication (PACS) radiology cloud server. All participant identifiers will be removed from x-rays prior to upload.

A panel of seven radiologists will each - independently and blinded to participant characteristics, HIV status, and results of microbiological investigations - classify chest x-rays using a standardised form for classification of chest radiology findings. Radiologists will review chest radiographs, and using an online data entry form, indicate the presence of any:

 - Infiltrate or consolidation

 - Cavitary lesion

 - Nodule or mass with poorly defined margins

 - Hilar/mediastinal adenopathy

 - Pleural effusion

 - Milliary findings

 - Discrete linear opacity

 - Discrete nodule(s) without calcification

 - Other findings

Radiologists will additionally classify chest radiographs as either suggestive of active pulmonary tuberculosis, or not. We will use the kappa statistic (two outcomes, multiple readers) with 95% confidence intervals to assess inter-reader agreement among the radiologists.

For the diagnostic accuracy evaluation, the index test will be CAD4TB score (continuous variable ranging from 0 to 100, and in a secondary analysis, dichotomised for greater than or equal to the CAD4TB threshold score of 45).

Reporting will follow the STARD Guidelines. For each index test definition (continuous distribution, and dichotomised to high vs. low probability of tuberculosis), we will compare diagnostic accuracy against two pre-defined reference standards:

1) Consensus radiologist classification with at least 5/7 independent readers agreeing that the radiograph was "suspicious of tuberculosis" (with sensitivity analysis limited to cases only where all 7/7 readers agreed), and
2) Bacteriologically-confirmed pulmonary tuberculosis, defined as either a documented positive GeneXpert result for *Mycobacterium tuberculosis* on at least one sample of sputum taken for study purposes at Day 56 assessment; or documented growth of *Mycobacterium tuberculosis* and positive speciation using MPT 64 antigen tests on at least one culture of sputum taken for study purposes at Day 56 assessment; or documented identification of acid fast bacilli on at least one sputum sample taken for study purposes and examined by sputum smear microscopy at Day 56 assessment.

For each comparison, sensitivity, specificity, positive predictive value, negative predictive value, and diagnostic odds ratios will be reported. Additionally, by constructing logistic regression models, we will investigate the effect of reader characteristics (practicing in Africa or elsewhere, years of practice) on diagnostic accuracy with bacteriological-confirmation as the reference standard.

## Validation of urinary lipoarabinomannan testing (LAM)

Urine LAM testing is a relatively new tuberculosis diagnostic, that has high accuracy among adults with advanced HIV infection, and has been shown to reduce mortality in hospitalised HIV-positive adults ^9^. The test is based on a lateral flow assay, and has been constructed to be used as a point of care test, with results read at the bedside.

However, sensitivity is known to be suboptimal among ambulant TB suspects. A newer version of the urine LAM test (FIND/Fujifilm) has been reported to have high sensitivity for TB, even among ambulant adults, and HIV-negative individuals. Therefore, the PROSPECT Study offers opportunity to undertake a nested evaluation of the performance of this test. We will collect a 5ml sample of urine from all participants at baseline, and transport the sample to the TB laboratory at the College of Medicine of Malawi for urine LAM testing. We will compare the diagnostic yield of urine LAM testing with that of sputum culture, smear and Xpert from day 56 participant samples.

### Data handling and management

Data will be collected by research assistants using the mobile CommCare data collection platform running on fingerprint secured tablets. Data will be transmitted to the secure study server over encrypted cellular networks. The MLW Data Department has considerable experience in building robust electronic data collection surveys and in secure data management, backup and processing. A full audit trial of database changes will be maintained.

Building upon our extensive experience of conducting previous trials using electronic data collection systems in Blantyre, the trial statistician and Chief Investigator will write scripts within the statistical programme R that will interface with the trial database and, on a regular automated basis, use logical rules to identify records with missing or implausible values that will be hand-checked against source records to ensure completeness and validity of the final dataset.

We are strongly committed to ensuring that the trial datasets are made openly available, and that all code used in the analysis are published to allow fully reproducible research. The data collected by this research will be of importance to other researchers and the public, and could for example be used by other researchers conducting meta-analysis, or by policymakers modelling the potential return on investment of implementing interventions within their settings. Therefore, we will establish a public online GitHub repository, where the final anonymised individual- level trial dataset and code to allow reproduction of all analysis will be published. The availability of these resources will be publicised within academic manuscripts, through the MLW and LSTM websites.

### Economic analysis

Two economic evaluations will be undertaken: firstly, a within-trial evaluation; and secondly, a decision-analytic based cost effectiveness model. Both will be used to estimate the expected incremental cost per quality-adjusted life year (QALY) gained for the two optimized TB/HIV interventions in comparison to standard of care. For both analyses, the perspective will be that of the Malawi Ministry of Health, and will only include the direct medical costs.

The within trial evaluation will adopt a time horizon matching the length of follow-up in the trial. The model-based evaluation will adopt a lifetime horizon so as to incorporate the long-term costs and health consequences of delayed TB/HIV diagnosis and treatment initiation.

For the within-trial evaluation, total costs and health benefits (QALYs) will be calculated over 56 days for each participant in each trial arm. Healthcare resource utilisation (e.g. clinic visits; investigations; medications) will be recorded over the 56 days from randomisation. Unit costs for these healthcare resources will be derived from primary costing studies, previous costing studies in Malawi or from targeted literature searches and inflated to the year of analysis. Unit costs for medications will be taken from the Management Sciences for Health International Drug Price Indicator guide. Responses to the EQ-5D-3L will be converted to health state utility values using the Zimbabwean tariff set and combined with the time spent within each health state to generate QALYs.

As the distributions of costs and QALYs are commonly skewed, and often bimodal or truncated, a range of estimators will be explored, and model diagnostics will be undertaken to determine optimal choice. Mean costs and outcomes for each intervention will be estimated, together with the mean incremental cost-effectiveness ratio. Measures of uncertainty (standard errors and confidence intervals) will also be reported for the mean estimates. The ICER will be calculated by comparing the least costly trial arm to the next least costly arm and calculated as below:

                       ICER = (Cost _2_ – Cost _1_)/(QALY _2_ – QALY _1_) 

Alternative trial arms that are more costly and less effective will be interpreted as dominated and would not represent an efficient use of resources. For alternative trial arms that are more costly and more effective, the interpretation of cost-effectiveness depends on the policy makers willingness to pay threshold (WTP) for a gain in QALY. Malawi and the majority of other African countries do not have an explicit WTP thresholds for interpreting cost-effectiveness. Hence cost-effectiveness acceptability curves (CEACs) will be constructed to identify the optimal intervention at different WTP thresholds.

The model-based evaluation will aim to extrapolate trial findings to allow estimation of cost-effectiveness over a lifetime time horizon. The model will likely consist of mutually exclusive Markov health states. These health states will be defined by a combination of untreated and treated states for both HIV and TB. The model will be parametrised by findings observed in the trial, and data extracted from the published literature.

### Translating research into policy

Results of this research will be important in guiding national, regional and international health policy. WHO, policymakers and parliamentarians are currently grappling with how to improve access to TB and HIV diagnosis and treatment, including the role of chest x-ray. A key objective of this study is therefore to translate research findings into normative guidance in Malawi, in sub-Saharan Africa, and through WHO.

We recognise that early engagement with policymakers is essential to translate research into action. Therefore, we undertaken preliminary scoping activities to identify key stakeholders that we will work with, including Malawi Ministry of Health TB/HIV Technical Working Groups, the Malawi Network for Evidence-Informed Decision Making (EvIDeNt) which includes regional linkage through the African Institute for Development Policy (AFIDEP), and WHO TB-STAG.

### Ethical considerations

This research has been approved by the College of Medicine of Malawi Research Ethics Committee (COMREC – number: P.11/17/2311), and the Research Ethics Committee of the Liverpool School of Tropical Medicine (number: 17-050). Trial progress will be reviewed by a data and safety monitoring board.

All prospective participants will be asked to provide written informed consent to take part in the trial. Individuals who are illiterate will be asked to provide a witnessed thumbprint to confirm their informed consent to participate. Witnesses will be an independent individual not involved with the study.

### Timelines

Piloting and preparatory activities will commence in April 2018, and trial recruitment in November 2018. We anticipate recruiting participants over an 8-month period, with final outcome assessment of treatment outcomes and mortality conducted after 6-months. Thus, the study will be completed in August 2020.

### Trial registration

This trial was registered with ClinicalTrials.gov on the 8 ^th^ May 2018 ( NCT03519425).

## Discussion

The PROSPECT Study will use a pragmatic trial design ^31^ to evaluate optimised TB/HIV screening and treatment linkage interventions under “real-life” condition in primary care in Malawi. The three-arm design allows us to efficiently test two important hypotheses.

Firstly, by comparing Group 2 to Group 1, we will investigate whether HIV care should be prioritised for adults with symptoms of TB. In previous studies, we and others have found operationalising universal HIV testing for adults attending primary care challenging due to limited counsellor capacity ^8^, meaning that only individuals in whom clinicians had a high suspicion of HIV were referred to the counsellor for HIV testing ^32^. Implementing a semi-supervised HIV self-testing intervention could then free-up counsellor capacity, increasing testing coverage. Moreover, self-testing is popular with patients, as it allows them to take control of the manner in which they learn their HIV status ^25, 33^. In addition to ensuring that individuals with TB symptoms are not “caught” between the HIV and TB care clinics ^16^, facilitated linkage to HIV care may promote earlier, more intensive screening for TB than would have otherwise occurred in the general outpatient clinic. Initiation of antiretroviral therapy may also unmask TB in individuals with advanced immunosuppression ^34^, prompting an earlier clinical decision to initiate tuberculosis treatment.

By comparing Group 3 to Group 2 and Group 1, we will provide strong evidence on the effectiveness of a novel triage approach to TB screening. As no single TB diagnostic currently has optimal characteristics in terms of test accuracy, reliability, implementation and scalability at primary care level, and cost per case detected, a triage approach that comprises an efficient, high sensitivity initial test, followed by a high specificity confirmatory test is required for patients with symptoms of TB. WHO recently undertook a modelling exercise to compare potential triage testing approaches, and found that, among adults with TB symptoms, an algorithm comprised of an initial chest x-ray followed by a confirmatory GeneXpert MTB/Rif test would likely fulfil requirements of having a high overall sensitivity and specificity, low number needed to screen to detect a case, and low cost per case detected ^22^. Moreover, this approach is attractive as, with the increasing availability of affordable digital x-ray units, the entire triage algorithm can be completed on the same day and within the same clinic. As an initial screening tool, chest x-ray can be performed quickly for large numbers of cases, screening out those with a low probability of disease. However, until now, widespread implementation has been limited by cost and by the availability of trained radiologists to classify x-rays. Therefore, in this study, we will evaluate the effectiveness of the CAD4TB computer aided diagnosis software system as the chest x-ray reader, which has demonstrated high accuracy (comparable to radiologists and clinicians) in diagnostic accuracy studies in Europe and Africa ^23^. In a nested evaluation, we will examine the diagnostic accuracy of the CAD4TB system compared to a panel of radiologists and sputum culture for *Mycobacterium tuberculosis.*

There are some limitations to this study. Although we will take extensive steps to minimise contamination between groups, including by participant fingerprint validation prior to intervention delivery, undertaking the trial in a single primary health may influence routine care decisions made by clinicians. There is a possibility of a Hawthorne effect, which might reduce the size of the differences between the standard of care arm and the interventions arms, and thus power might be reduced. Should evidence for effectiveness of these interventions be found, further supportive evidence would be provided by a future trial that randomly allocated clinics to intervention groups, and by surveillance of key process indicators under routine implementation conditions. The primary trial outcome will compare the time to TB treatment initiation between groups, an important indicator of individual and public health effectiveness. However, future larger studies may wish to investigate effectiveness against mortality, here investigated as a secondary outcome. Finally, as a sputum sample for TB culture will not be taken from participants in all groups at baseline, we will not be able to estimate the effectiveness of interventions on participants with true microbiologically-confirmed disease. This was a deliberate design of the study; TB culture is not widely-available in Africa as standard of care, meaning that empirical TB treatment is common ^35^. Should we have offered participants TB culture of sputum at baseline, we would not be able to obtain a true estimate of the effectiveness of interventions under “real-life” conditions. To ensure all participants are provided with high-quality screening and care, at Day 56 they will all be offered TB and HIV screening and supported to access care.

An important component of the PROSPECT Study is the evaluation of effects on patient-important outcomes – as measured by change in health-related quality of life – and cost-effectiveness. In Blantyre, we have well-established systems for patient and health resource costing ^36^, and will use both a within-trial cost-effectiveness evaluation, as well as a decision-analytic based cost effectiveness model. Additionally, we will work through the Policy Unit at the Malawi-Liverpool-Wellcome Trust Clinical Programme to engage early with national TB and HIV programmes in Malawi, and with regional and supranational policy fora. The exploratory Bayesian trial analysis will incorporate the prior beliefs of various groups of stakeholders to ensure that meaningful evidence can be provided to key groups.

## Dissemination of findings

Trial results and findings from the cost-effectiveness analysis will be shared with Blantyre District Health Office, the Malawi National Tuberculosis Programme and with the Malawi National HIV Programme. We will report findings at national, regional and international conferences, and will submit a manuscript reporting trial findings to a peer-reviewed journal specialising in public health, HIV and tuberculosis.

To facilitate reproducibility of analysis, an anonymised minimal final dataset and all code required to reproduce analysis will be published in the trial GitHub repository.

## Current trial status

The trial is currently in preparatory and piloting phase.

In summary, the PROSPECT Study will provide urgently-needed evidence under “real-life” conditions to inform clinicians and policy makers on how best to improve TB/HIV diagnosis and treatment initiation in Africa.

## Data availability

All data underlying the results are available as part of the article and no additional source data are required.

## Supplementary material

Supplementary File 1 – Baseline questionnaire.

Supplementary File 2 – Statistical analysis plan.
