# Supplementary material for: Design and protocol for a pragmatic randomised study to optimise screening, prevention and care for tuberculosis and HIV in Malawi (PROSPECT Study)
Source: Wellcome Open Res. 2018 Nov 21;3:61. Originally published 2018 May 18. [Version 3] doi: 10.12688/wellcomeopenres.14598.3 (PMC6259593; doi:10.12688/wellcomeopenres.14598.3)
Supplement: Supplementary file 1 [file wellcomeopenres-3-16287-s0000.tgz › e47fad21-99db-4d55-8ec1-57e5a5f044a7.docx]

| **A pragmatic randomised study to optimise screening, prevention and care for tuberculosis in Malawi (The PROSPECT Study)** |
| --- |
| **Principal Investigator: Dr Peter MacPherson Malawi-Liverpool-Wellcome Trust Clinical Research Programme PO Box 30096, Chichiri, Blantyre 3, Malawi Tel +265 1 874 628 www.mlw.medcol.mw** |

CRF (Blank English: COMMCARE VERSION: 1031

#### [Registration](file:////a/prospect-1/apps/view/2a235d4096443d62135df383d58bb999/module/d5272758ea2be67a86d94bbf98e725baff0e7e03/)

##### [A Eligibility and consent](file:////a/prospect-1/apps/view/2a235d4096443d62135df383d58bb999/form/34e1c6e448d30c0a10e661342cfc51351c1123df/)

- - 1. This form should be completed for all adults attending the health centre with an acute care episode. This form determines whether they are eligible to take part in the PROSPECT Study.
    2. Enter participant's first and surname (for example "John Banda") *
    3. Are you male or female? *
       1. Male
       2. Female
    4. What is your current age in years? *
    5. Mobile number 1 *
    6. Please re-enter the participant's mobile phone number *
    7. [unknown]
    8. Did participant agree to have fingerprints captured? *
       1. Yes
       2. No
    9. Do you have a cough now? *
       1. Yes
       2. No
    10. Have you been losing weight? *
        1. Yes
        2. No
    11. Do you have night sweats? *
        1. Yes
        2. No
    12. Do you have fever or hot body? *
        1. Yes
        2. No
    13. This individual is not eligible to take part in the PROSPECT Study. DO NOT COMPLETE ANY MORE FORMS. Thank them for their time, and refer them to the clinic waiting room.
    14. Are you on TB treatment? By TB treatment I mean registered at one of the TB clinics and receiving 6-8 months of treatment with a TB card? *
        1. Yes
        2. No
    15. Have you taken treatment for TB in the last 6-months *
        1. Yes
        2. No
    16. Are you currently taking isoniazid preventive therapy? *
        1. Yes
        2. No
    17. Do you live in Blantyre? *
        1. Yes
        2. No
    18. Do you plan to move out of Blantyre to live elsewhere in the next 6-months? *
        1. Yes
        2. No
    19. This individual is not eligible to take part in the PROSPECT Study. DO NOT COMPLETE ANY MORE FORMS. Thank them for their time and refer them to the waiting area.
    20. This participant is ELIGIBLE to take part in the study. NOW complete the consent form
    21. Did this individual provide informed voluntary consent to participate in the PROSPECT Study? *
        1. Yes
        2. No
    22. As participant DID NOT consent to participate, they cannot be recruited to the study. Thank them for their time and refer to the clinic waiting room.
    23. PLEASE COMPLETE THE STUDY PARTICIPANT LOG. Record Participant Log ID: (e.g. 0001) *
    24. #form/a0081_elig1
    25. #form/rightThumb
    26. #form/a0131_elig2
    27. #form/a0150_recruited
    28. #form/a0080_cghelig
    29. #form/simprintsId
    30. #form/rightIndex
    31. #form/leftThumb
    32. #form/a0010_indate
    33. #form/leftIndex

#### [Case List](file:////a/prospect-1/apps/view/2a235d4096443d62135df383d58bb999/module/b64ad6d1e40600d61ed323ec31b869776a7df4fe/)

##### [B Baseline characteristics](file:////a/prospect-1/apps/view/2a235d4096443d62135df383d58bb999/form/9bd0b67e8a470f7e05e80658d2e21eaad469db98/)

- - 1. This form should be completed for adults who are eligible and have consented to take part in the PROSPECT Study. If you have selected this form in error, press HOME.
    2. What is your date of birth? *
    3. Are you currently pregnant? *
       1. Yes
       2. No
    4. What is the highest level of education you have completed? *
       1. Never been to school
       2. Primary
       3. Secondary no MSCE
       4. Secondary completed MSCE
       5. Higher
    5. Can you read a newspaper or a letter? *
       1. Yes
       2. No
    6. How would you best describe your main activity or work status? *
       1. Paid employee
       2. Paid domestic worker
       3. Self-employed
       4. Unemployed
       5. Student
       6. Other
    7. How do you rate your general health? *
       1. Very good
       2. Good
       3. Fair
       4. Poor
       5. Very poor
    8. What is your current marital status? (probe to get accurate answer) *
       1. Married
       2. Polygamous marriage
       3. Living together as if married
       4. Never married
       5. Widowed
       6. Seperated
       7. Divorced
       8. Married but not living together
    9. Have you ever lost a spouse to death? *
       1. Yes
       2. No
    10. Have you ever been tested for HIV? *
        1. Yes
        2. No
    11. In the last 12 months, how many times have you tested for HIV? *
    12. You don't have to tell me if you don’t want to, but what was the result of your last HIV test? *
        1. Positive
        2. Negative
        3. Don't know
        4. Don't want to reveal
        5. Never tested
    13. Have you ever had a positive HIV test? *
        1. Yes
        2. No
    14. Are you currently taking ART drugs? *
        1. Yes
        2. No
    15. Have you ever taken ART drugs? *
        1. Yes
        2. No
    16. When did you start taking ART?
    17. Which ART medication are you currently taking? *
        1. Regimen 0 (ABC + 3TC + NVP)
        2. Regimen 1 (D4T + 3TC + NVP)
        3. Regimen 2 (AZT + 3TC + NVP)
        4. Regimen 4 (AZT + 3TC + EFV)
        5. Regimen 5 (TDF + 3TC + EFV)
        6. Regimen 6 (TDF + 3TC + NVP)
        7. Regimen 7 (TDF + 3TC + ATV/r)
        8. Regimen 8 (AZT + 3TC + ATV/r)
        9. Regimen 9 (ABC + 3TC + LPV/r)
        10. Regimen 10 (TDF + 3TC + LPV/r)
        11. Regimen 11 (AZT + 3TC + LTV/r)
        12. Regimen 12 (DRV + r + ETV + RAL)
        13. Other
    18. Are you taking co-trimoxazole preventive therapy (CPT)? *
        1. Yes
        2. No
    19. Date co-trimoxazole preventive therapy (CPT) started? *
    20. Have you ever been treated for TB? *
        1. Yes
        2. No
    21. When did you start the TB treatment? *
    22. Do you have a cough now? *
        1. Yes
        2. No
    23. How long have you been coughing? If less than 12 months, answer must be recorded in days, weeks or months. If 12 months (1 year or more) answer must be recorded in years) *
    24. Units of cough duration *
        1. Days
        2. Weeks
        3. Months
        4. Years
    25. Do you have night sweats? *
        1. Yes
        2. No
    26. Are you losing weight? *
        1. Yes
        2. No
    27. Do you have a fever or hot body? *
        1. Yes
        2. No
    28. Mobility: Which statements best describe your own health state today? *
        1. I have no problems in walking about
        2. I have some problems in walking about
        3. I am confined to bed
    29. Self-care: Which statements best describe your own health state today? *
        1. I have no problems with self-care
        2. I have some problems washing or dressing myself
        3. I am unable to wash or dress myself
    30. Usual Activities (e.g. work, study, housework, family or leisure activities): Which statement best describe your own health state today? *
        1. I have no problems with performing my usual activities
        2. I have some problems with performing my usual activities
        3. I am unable to perform my usual activities
    31. Pain/Discomfort: Which statements best describe your own health state today? *
        1. I have no pain or discomfort
        2. I have moderate pain or discomfort
        3. I have extreme pain or discomfort
    32. Anxiety/Depression: Which statements best describe your own health state today? *
        1. I am not anxious or depressed
        2. I am moderately anxious or depressed
        3. I am extremely anxious or depressed
    33. To help people say how good or bad a health state is, we have drawn a scale (rather like a thermometer) on which the best state you can imagine is marked 100 and the worst state you can imagine is marked 0. We would like you to indicate on this scale how good or bad your own health is today, in your opinion. Please do this by drawing a line from the box below to whichever point on the scale indicates how good or bad your health state is today. *
    34. Height (in cm) *
    35. Weight (in kg) *
    36. #form/b9999_complete

##### [C Locator form](file:////a/prospect-1/apps/view/2a235d4096443d62135df383d58bb999/form/e1b1e9248c682fd24a4fbe2e28846c9baea8d7f7/)

- - 1. This form should be completed for all participants recruited to the PROSPECT Study. This form will help to trace participants if they don't return for follow-up. If you have selected this form in error, please press BACK.
    2. Please record detailed instructions about how to find the participant's house. Should be able to find the house easily by following these instructions. (e.g: Ndirande, New Lines, Four ways House number 575 kwa a Juma) *
    3. Enter all mobile numbers that we can use to contact you. (Mobile number 1) *
    4. Enter all mobile numbers that we can use to contact you. (Mobile number 2)
    5. Enter all mobile numbers that we can use to contact you. (Mobile number 3)
    6. What are the best days to visit you at home if we need to? *
       1. Monday
       2. Tuesday
       3. Wednesday
       4. Thursday
       5. Friday
       6. Saturday
       7. Sunday
    7. What are the best times to visit you at home if we need to? *
       1. 07:00-09:00
       2. 09:00-12:00
       3. 12:00-15:00
       4. 15:00-17:00
    8. #form/c9999_complete

##### [D Randomisation](file:////a/prospect-1/apps/view/2a235d4096443d62135df383d58bb999/form/83cab3163338aeb0c9db2232949d38e3588bc41/)

- - 1. This form should be completed to randomly allocate participants to one of the three intervention groups in the PROSPECT Study. Participants can only be randomised once. If you have selected this form in error, please press HOME.
    2. This participant has the following randomisation ID: ____
    3. This participant has been allocated to: ____
    4. Record the Day 56 follow-up appointment date in the appointment register, then enter here. *
    5. Record the Day 56 appointment time in the Study Appointment Register, then enter here. *
    6. #form/d9999_complete
    7. #form/d0011_randout
    8. #form/user_prefix
    9. #form/visit_count
    10. #form/id_for_group

##### [E HIV testing episode](file:////a/prospect-1/apps/view/2a235d4096443d62135df383d58bb999/form/878e4a7e4b9e4841fb24ead84530189d2762d154/)

- - 1. This form should be completed for any participant undergoing a study HIV testing episode.
    2. What is the reason for this HIV test? *
       1. Baseline HIV testing episode
       2. Repeat baseline HIV testing episode
       3. Day 56 outcome HIV testing episode
       4. Repeat Day 56 testing episode
    3. Is this participant known to be HIV-positive and taking ART? *
       1. Yes
       2. No
    4. As this participant is known to be HIV-positive and taking ART, do not perform HIV testing today. Check their status as "HIV-positive" on the next screen.
    5. This person is not eligible to receive a baseline study HIV test. Please refer them to the clinic waiting area to be seen by routine clinic health workers.
    6. This participant is eligible for HIV testing today
    7. As part of this study, we are offering you the opportunity to take an HIV test now. HIV is the virus that causes AIDS. AIDS is a very serious illness. There is good treatment now available for AIDS that will help improve your health and stop you passing the HIV virus to others. If we find that you have HIV, we will make sure that you get the treatment that you need. For the HIV test, we need a mouth swab that may be followed with a fingerprick. The equipment used to take the blood is clean and completely safe. It has never been used before and will be thrown away after each test.
    8. Do you agree to do the HIV test? *
       1. Yes
       2. No
    9. As this participant has agreed to HIV testing, please perform OraQuick testing according to SOP
    10. Do you agree for me to do the test, but to not tell you the result? *
        1. Yes
        2. No
    11. As this participant has agreed to anonymous HIV testing for study purposes, please perform OraQuick HIV testing, but DO NOT inform participant of the result. Provide them with generic post test HIV counselling. Record the result as HIV-positive or HIV-negative on the final page.
    12. Result of anonymous OraQuick HIV test for study purposes *
        1. Reactive
        2. Non-reactive
    13. Result of OraQuick HIV test *
        1. Reactive
        2. Non-reactive
    14. As this participant's OraQuick test was NON-REACTIVE, they can be given a result of HIV-NEGATIVE. Please provide post-test HIV prevention counselling
    15. As this participant's OraQuick test was REACTIVE, please perform confirmatory testing using DETERMINE 1/2 according to SOP
    16. Result of Determine 1/2 HIV test *
        1. Reactive
        2. Non-reactive
    17. As this participant's OraQuick and Determine 1/2 HIV tests results are both REACTIVE, they can be provided with a result of HIV-POSITIVE. Please provide post-test HIV counselling.
    18. Result of repeat OraQuick HIV test *
        1. Reactive
        2. Non-reactive
    19. Result of repeat Determine 1/2 HIV test *
        1. Reactive
        2. Non-reactive
    20. Result of Unigold HIV test *
        1. Reactive
        2. Non-reactive
    21. We have not been able to determine you HIV status today. This means that we need to repeat these tests in 2 weeks time. MAKE AN APPOINTMENT FOR REPEAT TESTING IN 2-WEEKS
    22. Date and time of appointment for repeat HIV testing. *
    23. Final HIV status *
        1. HIV-positive
        2. HIV-negative
        3. Inconclusive
        4. Not tested
    24. As this participant is HIV-positive, please register them for care at the HIV care clinic. Complete Form K when registering the participant for HIV care and ART.
    25. #form/e0011_baselinegroupelig
    26. #form/e0071_tiebreaker1
    27. #form/e0012_baselineeligtype
    28. #form/e0015_futest
    29. #form/e0013_baselinecantest
    30. #form/e0090_incon
    31. #form/e0072_tiebreaker2

##### [F Baseline TB screening episode](file:////a/prospect-1/apps/view/2a235d4096443d62135df383d58bb999/form/f1c8c673bb62f3eecf6288530c8202d30eae1fe0/)

- - 1. This participant is not eligible for study TB investigations. Please refer them to the clinic waiting are to be assessed by routine clinic health workers.
    2. As part of this study, we would like to take a picture of your chest using an x-ray to help see if you have tuberculosis. This is safe, and you will not feel anything during the x-ray procedure.
    3. Confirm chest x-ray completed *
       1. Yes
       2. No
    4. What is the CAD4TB score? *
    5. As participants CAD score is less than XX, they do not require any further study TB tests today. Please refer them to the clinic waiting area to be seen by the routine clinic health workers. Remind them to return to the clinic for reassessment if they are not getting better. Remind them to attend their booked DAY 56 appointment.
    6. As participant's CAD score is greater than XX, they require further study sputum tests to confirm or rule out tuberculosis.
    7. Please provide the participant with instructions on producing high quality sputum samples according to SOP. Ensure that sputum is produced in a well-ventilated, outdoors space.
    8. Was sputum induction using saline required? *
       1. Yes
       2. No
    9. Was sputum sample successfully collected? *
       1. Yes
       2. No
    10. As baseline sputum sample could not be collected, please provide the participant with a sputum cup to take home, ask them to produce sputum into the cup first thing tomorrow morning, and bring back to the study office.
    11. Confirm that sputum sample has been analysed by GeneXpert *
        1. Yes
        2. No
    12. GeneXpert results *
        1. M Tuberculosis detected
        2. M Tuberculosis not detected
        3. Not done
    13. GeneXpert Rifampacin resistance result *
        1. Rifampacin resistance NOT DETECTED
        2. Rifampacin resistance DETECTED
    14. GeneXpert cycle threshold *
    15. As participant has a positive GeneXpert result, please inform them that they have microbiologically-confirmed pulmonary tuberculosis and need treatment. Register them for tuberculosis treatment at the clinic. Complete Form L when registering them for TB treatment.
    16. Please inform this participant that their investigations for tuberculosis are negative (or have not been able to be completed today), and they do not require treatment at this time. However, inform them that should they continue to have symptoms, they should return to the routine clinic for assessment. Refer the participant to the waiting room to be seen by the routine facility health worker. Remind the participant to return for their booked DAY 56 assessment.
    17. #form/f0007_cadpos

##### [G Day 56 Questionnaire](file:////a/prospect-1/apps/view/2a235d4096443d62135df383d58bb999/form/181ea393c6e4db6709a978efac7bda79f0e3e482/)

- - 1. How do you rate your general health? *
       1. Very good
       2. Good
       3. Fair
       4. Poor
       5. Very poor
    2. Do you have a cough now? *
       1. Yes
       2. No
    3. How long have you been coughing? If less than 12 months, answer must be recorded in days, weeks or months. If 12 months (1 year or more) answer must be recorded in years) *
    4. Units of cough duration *
       1. Days
       2. Weeks
       3. Months
       4. Years
    5. Do you have night sweats? *
       1. Yes
       2. No
    6. Are you losing weight? *
       1. Yes
       2. No
    7. Do you have a fever or hot body? *
       1. Yes
       2. No
    8. Are you taking Isoniazid Preventive Therapy (IPT)? *
       1. Yes
       2. No
    9. Date Isoniazid preventive therapy (IPT) started *
    10. Are you taking co-trimoxazole preventive therapy (CPT)? *
        1. Yes
        2. No
    11. Date co-trimoxazole preventive therapy (CPT) started? *
    12. Are you taking TB treatment now? By TB treatment I mean registered at one of the TB clinics and receiving 6-8 months of treatment with a TB card? *
        1. Yes
        2. No
    13. How was TB treatment confirmed? (Check all that apply) *
        1. Inspected TB treatment card
        2. Inspected facility TB register
        3. Inspected TB medication/tablets
    14. Which TB treatment centre did participant register for treatment at? *
        1. Blantyre Adventist Hospital (BAH)
        2. Bangwe (BG)
        3. BTGW
        4. Chilomoni (CH)
        5. Chitawira (CW)
        6. Gateway Clinic (GW)
        7. Limbe Health Centre (LB)
        8. Mlambe (MB)
        9. Mwaiwathu (MW)
        10. Ndirande (ND)
        11. Queen Elizabeth Central Hospital (QE)
        12. Queen Elizabeth Central Hospital 2 (QE2)
        13. South Lunzu (SL)
        14. Zingwangwa (ZW)
        15. Other
    15. TB Registration number *
    16. Date of TB registration *
    17. Date TB treatment started *
    18. Tuberculosis disease site (check one) *
        1. Pulmonary
        2. Extra-Pulmonary
    19. Sputum smear result *
        1. Sputum smear positive
        2. Sputum smear negative
        3. Not done
    20. Xpert MTB/Rif results *
        1. MTB detected
        2. MTB not detected
        3. Not done/laboratory problem
    21. Xpert MTB/Rif resistance testing result *
        1. RIF resistance detected
        2. RIF Resistance not detected
        3. Not done/laboratory problem
    22. Chest x-ray result *
        1. Chest x-ray suggestive of TB
        2. CXR not suggestive of TB
        3. Not done
    23. TB culture result *
        1. TB culture positive
        2. Culture_negative
        3. Not done
    24. Type of TB patient (check one) *
        1. New
        2. Relapse
        3. Transfer in
        4. Treatment after default
        5. Treatment after failure
        6. Other
    25. Initial TB treatment phase drugs (select all that apply) *
        1. RHZE
        2. S
        3. Other
    26. Continuation Phase TB treatment drugs (select all that apply) *
        1. RH
        2. RHE
        3. Other
        4. Not yet on continuation phase
    27. Date TB treatment last dispensed *
    28. Have you ever been tested for HIV? *
        1. Yes
        2. No
    29. What was the results of your last (most recent) HIV test? *
        1. HIV-positive
        2. HIV-negative
        3. Never previously tested
    30. Have you ever had a positive HIV test? *
        1. Yes
        2. No
    31. Are you currently taking ART drugs for treatment of HIV? *
        1. Yes
        2. No
    32. Participant's ART card inspected and confirmed taking ART treatment? *
        1. Yes
        2. No
    33. Date ART started *
    34. Date ART last dispensed *
    35. Which ART medication are you currently taking *
        1. Regimen 0 (ABC + 3TC + NVP)
        2. Regimen 1 (D4T + 3TC + NVP)
        3. Regimen 2 (AZT + 3TC + NVP)
        4. Regimen 3 (D4T + 3TC + EFV)
        5. Regimen 4 (AZT + 3TC + EFV)
        6. Regimen 5 (TDF + 3TC + EFV)
        7. Regimen 6 (TDF + 3TC + NVP)
        8. Regimen 7 (TDF + 3TC + ATV/r)
        9. Regimen 8 (AZT + 3TC + ATV/r)
        10. Regimen 9 (ABC + 3TC + LPV/r)
        11. Regimen 10 (TDF + 3TC + LPV/r)
        12. Regimen 11 (AZT + 3TC + LTV/r)
        13. Regimen 12 (DRV + r + ETV + RAL)
        14. Other
    36. Since we recruited you to this study (Day 0: ____) and today, have you been admitted to hospital for any reason? By "admitted to hospital", I mean stayed at least one night in hospital*
        1. Yes
        2. No
    37. Since we recruited you to this study (Day 0: ____) and today, how many times were you admitted to hospital? By this, I mean how many episodes did you have when you stayed in hospital for at least one night. *
    38. Hospital admission episode number
    39. You have told me that you were admitted to hospital a total of ____ times. I am now going to ask you about episode number: ____
    40. On what date did this hospital admission occur? *
    41. During this admission, how many nights did you spend in hospital in total? *
    42. During this admission, what was the reason for your admission? *
        1. Anaemia
        2. Thrush in the mouth or throat
        3. Stroke
        4. Diarrhoea (sudden, or with blood)
        5. Diarrhoea (that has gone on for a long time)
        6. Heart failure
        7. Cryptococcal meningitis
        8. Diabetes (with complications)
        9. Diabetes (without complications)
        10. Diseases of the kidneys, bladder or womb
        11. Muscle or bone problems
        12. Epilepsy / seizures / convulsion
        13. High blood pressure (hypertension)
        14. Kaposi's sarcoma
        15. Liver disease
        16. Malaria
        17. Meningitis
        18. Mental health problems
        19. Cancers (excluding Kaposi's sarcoma)
        20. Other heart (cardiovascular problems)
        21. Other brain or nerve (neurological) problems
        22. Other breathing/lung problems
        23. Pneumocystis Jivorecii Pneumonia (PCP)
        24. Pneumonia (infection of the lungs)
        25. Pregnancy / maternity related
        26. TB of the lungs (pulmonary TB)
        27. Blood infection (scepticaemia)
        28. Tuberculosis (retreatment)
        29. Tuberculosis of bones and joints
        30. Tuberculosis of the gut/stomach
        31. Tuberculosis of the brain / meningitis
        32. Miliary tuberculosis (TB throughout the whole body)
        33. Tuberculosis of any other organ
        34. Upper gastrointestinal problems (problems with the throat or stomach)
        35. Viral infection
        36. Any other reason
        37. Don't know/can't remember
    43. During this admission, did you have any sputum tests done for TB testing? (check all that apply) *
        1. Sputum smear
        2. GeneXpert test
        3. Sputum culture
        4. No sputum tests done
        5. Don't know
    44. During this admission, did you have any x-ray tests done? *
        1. Chest x-ray done
        2. Another x-ray done
        3. No x-rays done
        4. Don't know
    45. During this admission, did you start treatment for tuberculosis? *
        1. Yes
        2. No
        3. Don't know/can't remember
    46. During this admission, did you have an HIV test done? *
        1. Yes
        2. No
        3. Don't know/can't remember
    47. During this admission, did you start ART treatment for HIV? *
        1. Yes
        2. No
        3. Don't know/can't remember
    48. During this admission, did you have any blood tests done? *
        1. Yes
        2. No
        3. Don't know/can't remember
    49. During this admission, did you have any antibiotic injections? *
        1. Yes
        2. No
        3. Don't know/can't remember
    50. For how many days did you receive antibiotic injections? *
    51. Since we recruited you to this study (Day 0: ____), and today, have you attended any health centres or clinics? *
        1. Yes
        2. No
    52. Since we recruited you to this study (Day 0: ____) and today, how many times did you attend a health centre or clinic? *
    53. Clinic attendance episode number
    54. You have told me that you attended a clinic or health centre total of ____ times. I am now going to ask you about clinic attendance episode number: ____
    55. On what date did this clinic attendance occur? *
    56. On this clinic attendance, which clinic did you attend? *
        1. Bangwe (BG)
        2. Chilomoni (CH)
        3. Chitawira (CW)
        4. Gateway Clinic (GW)
        5. Limbe Health Centre (LB)
        6. Mlambe (MB)
        7. Mwaiwathu (MW)
        8. Ndirande (ND)
        9. Queen Elizabeth Central Hospital (QE)
        10. South Lunzu (SL)
        11. Zingwangwa (ZW)
        12. Other
    57. What was your main reason for attending the clinic on this occasion? *
        1. For HIV testing/counselling
        2. To collect antiretroviral therapy for treating HIV
        3. To attend HIV care clinic (not taking antiretroviral therapy)
        4. To register for, or collect, TB treatment
        5. Hypertension/high blood pressure clinic (routine follow-up)
        6. Diabetes clinic (routine follow-up)
        7. Antenatal clinic / maternity clinic
        8. Feeling unwell / wanted to get treatment
        9. Other reason
    58. During this clinic attendance, did you have any sputum tests done for TB testing? (check all that apply) *
        1. Sputum smear
        2. GeneXpert test
        3. Sputum culture
        4. No sputum tests done
        5. Don't know
    59. During this attendace, did you have any x-ray tests done? *
        1. Chest x-ray done
        2. Another x-ray done
        3. No x-rays done
        4. Don't know
    60. During this attendance, did you start treatment for tuberculosis? *
        1. Yes
        2. No
        3. Don't know/can't remember
    61. During this admission, did you have an HIV test done? *
        1. Yes
        2. No
        3. Don't know
    62. During this attendance, did you start ART treatment for HIV? *
        1. Yes
        2. No
        3. Don't know/can't remember
    63. During this attendance, did you have any blood tests done? *
        1. Yes
        2. No
        3. Don't know
    64. During this attendance, did you receive any antibiotic tablets? *
        1. Yes
        2. No
        3. Don't know/can't remember
    65. For how many days did the health worker tell you to take the antibiotic tablets? *
    66. Mobility: Which statements best describe your own health state today? *
        1. I have no problems in walking about
        2. I have some problems in walking about
        3. I am confined to bed
    67. Self-care: Which statements best describe your own health state today? *
        1. I have no problems with self-care
        2. I have some problems washing or dressing myself
        3. I am unable to wash or dress myself
    68. Usual Activities (e.g. work, study, housework, family or leisure activities): Which statement best describe your own health state today? *
        1. I have no problems with performing my usual activities
        2. I have some problems with performing my usual activities
        3. I am unable to perform my usual activities
    69. Pain/Discomfort: Which statements best describe your own health state today? *
        1. I have no pain or discomfort
        2. I have moderate pain or discomfort
        3. I have extreme pain or discomfort
    70. Anxiety/Depression: Which statements best describe your own health state today? *
        1. I am not anxious or depressed
        2. I am moderately anxious or depressed
        3. I am extremely anxious or depressed
    71. To help people say how good or bad a health state is, we have drawn a scale (rather like a thermometer) on which the best state you can imagine is marked 100 and the worst state you can imagine is marked 0. We would like you to indicate on this scale how good or bad your own health is today, in your opinion. Please do this by drawing a line from the box below to whichever point on the scale indicates how good or bad your health state is today. *
    72. Height (in cm) *
    73. Weight (in kg) *
    74. Now complete FORM E (HIV Testing Episode) and FORM H (Day 56 TB Screening Episode)
    75. #form/g9999_complete
    76. #form/g0152_attendno/g01511_hidden
    77. #form/g0140_episode/g01401_hidden

##### [H Day 56 TB screening episode](file:////a/prospect-1/apps/view/2a235d4096443d62135df383d58bb999/form/577b41b11a94beeb970c5094d496e9e1b99d774a/)

- - 1. PREPERATION INSTRUCTIONS: * Detach three barcode stickers from the same row * Check barcode stickers all have the same number * Stick barcode sticker #1 to sputum cup 1 * Stick barcode sticker #2 to sputum cup 2 * Stick barcode sticker #3 to laboratory request form * Scan barcode using phone camera *
    2. INSTRUCTIONS (SHOW PARTICIPANT SPUTUM COLLECTION CARD) (READ TO PARTICIPANT): * We need to collect sputum coughed from your lungs to test whether you have TB. * The sputum will be tested in the lab * If your sputum test shows you have TB, we will contact you and help you get treatment * If you don't hear from us, that means you don't have TB * However, if you still don't feel well, you should come back to see the health worker at the clinic
    3. INSTRUCTIONS (READ TO PARTICIPANT) * We need to collect two cups of sputum coughed from your lungs. * We **don't** want saliva from your mouth. **1st Specimen now** * Move away from other patients to the outdoor area. * Rinse your mouth with some clean water. **Do this three times** * Slowly take a deep breath in. * blow out *fully and quickly* **Now take a deep breath and quickly cough up sputum from deep in your chest, into the pot** * Enough to fill a spoon is sufficient. * Put the lid on before you give it to us. **2nd Specimen - wait one hour** * Wait **1 hour** before the second specimen. *Move away from other patients to the outdoor area. * Rinse your mouth with clean water. * Repeat the breathing like you did before. * Take a deep breath in and out three times. * Cough sputum from your lungs into the pot. * Put the lid on before you give it to us. **If you are having difficulties giving sputum, tell the Research Assistant, who will help you.**
    4. Was sputum induction using saline required? *
       1. Yes
       2. No
    5. Were 2x sputum samples successfully collected? *
       1. Yes
       2. No
    6. **INSTRUCTIONS** * As Day 56 sputum samples could not be collected, please provide the participant with two sputum cups labelled with barcodes to take home. * Make sure the laboratory request form has the barcode sticker attached * Place the cups in a bag with the laboratory form, and give to the participant * Ask them to produce sputum into the cup first thing tomorrow morning, * Tell them to bring back the sputum cups in the bag with the form to the study office. **NOW: Record in study register that participant has not submitted sputum samples** * Undertake home tracing to collect sputum if participant doesn't return with samples
    7. Was participant given sputum cups to take home? *
       1. Yes
       2. No
    8. Inform participant that they will only be contacted if their TB sputum test result is positive. Thank them for participating in the study. REMEMBER TO COMPLETE FORM E (HIV TESTING EPISODE)
    9. #form/h9999_complete

##### [J Home tracing episode](file:////a/prospect-1/apps/view/2a235d4096443d62135df383d58bb999/form/c375aa6fc3750e17154308c2d668c77456ea3b9a/)

- - 1. What is the reason for home tracing visit? *
       1. Participant didn't attend Day 56 assessment
       2. Participant didn't return to clinic with sputum samples
       3. Other
    2. What home visit is this? *
       1. Visit 1
       2. Visit 2
       3. Visit 3
    3. Was participant (or a key informant) successfully traced? *
       1. Yes
       2. No
    4. As participant was not traced, return to Research Office and attempt further telephone contact and home tracing to establish location, up to a maximum of three home visits.
    5. Who was interviewed? *
       1. Participant
       2. Another household member
       3. Someone else from outside the household
    6. Is participant deceased? *
       1. Yes
       2. No
    7. On what date did the participant die? *
    8. Is (was) participant taking Isoniazid Preventive Therapy (IPT)? *
       1. Yes
       2. No
    9. Date Isoniazid preventive therapy (IPT) started *
    10. Is (was) participant taking co-trimoxazole preventive therapy (CPT)? *
        1. Yes
        2. No
    11. Date co-trimoxazole preventive therapy (CPT) started? *
    12. Is (was) participant taking TB treatment? By TB treatment I mean registered at one of the TB clinics and receiving 6-8 months of treatment with a TB card? *
        1. Yes
        2. No
    13. How was TB treatment confirmed? (Check all that apply)
        1. Inspected TB treatment card
        2. Inspected facility TB register
        3. Inspected TB medication/tablets
    14. Which TB treatment centre did participant register for treatment at? *
        1. Blantyre Adventist Hospital (BAH)
        2. Bangwe (BG)
        3. Chilomoni (CH)
        4. Chitawira (CW)
        5. Gateway Clinic (GW)
        6. Limbe Health Centre (LB)
        7. Mlambe (MB)
        8. Mwaiwathu (MW)
        9. Ndirande (ND)
        10. Queen Elizabeth Central Hospital (QE)
        11. Queen Elizabeth Central Hospital 2 (QE2)
        12. South Lunzu (SL)
        13. Zingwangwa (ZW)
        14. Other
        15. Don't know
    15. TB Registration number *
    16. Date of TB registration *
    17. Date TB treatment started *
    18. Tuberculosis disease site (check one) *
        1. Pulmonary
        2. Extra-Pulmonary
    19. Sputum smear result *
        1. Sputum smear positive
        2. Sputum smear negative
        3. Not done
        4. Don't know
    20. Xpert MTB/Rif results *
        1. MTB detected
        2. MTB not detected
        3. Not done/laboratory problem
        4. Don't know
    21. Xpert MTB/Rif resistance testing result *
        1. RIF resistance detected
        2. RIF Resistance not detected
        3. Not done/laboratory problem
        4. Don't know
    22. Chest x-ray result *
        1. Chest x-ray suggestive of TB
        2. CXR not suggestive of TB
        3. Not done
        4. Don't know
    23. TB culture result *
        1. TB culture positive
        2. Culture_negative
        3. Not done
        4. Don't know
    24. Type of TB patient (check one) *
        1. New
        2. Relapse
        3. Transfer in
        4. Treatment after default
        5. Treatment after failure
        6. Other
        7. Don't know
    25. Initial TB treatment phase drugs (select all that apply) *
        1. RHZE
        2. S
        3. Other
        4. Don't know
    26. Continuation Phase TB treatment drugs (select all that apply) *
        1. RH
        2. RHE
        3. Other
        4. Don't know
    27. Date TB treatment last dispensed *
    28. What was the results of participant's last (most recent) HIV test? *
        1. HIV-positive
        2. HIV-negative
        3. Never previously tested
        4. Don't know
    29. Has participant ever had a positive HIV test? *
        1. Yes
        2. No
        3. Don't know
    30. Is (was) participant taking ART drugs for treatment of HIV? *
        1. Yes
        2. No
        3. Don't know
    31. Participant's ART card inspected and confirmed taking ART treatment? *
        1. Yes
        2. No
    32. Date ART started *
    33. Date ART last dispensed *
    34. Which ART medication is (was) participant currently taking *
        1. Regimen 0 (ABC + 3TC + NVP)
        2. Regimen 1 (D4T + 3TC + NVP)
        3. Regimen 2 (AZT + 3TC + NVP)
        4. Regimen 3 (D4T + 3TC + EFV)
        5. Regimen 4 (AZT + 3TC + EFV)
        6. Regimen 5 (TDF + 3TC + EFV)
        7. Regimen 6 (TDF + 3TC + NVP)
        8. Regimen 7 (TDF + 3TC + ATV/r)
        9. Regimen 8 (AZT + 3TC + ATV/r)
        10. Regimen 9 (ABC + 3TC + LPV/r)
        11. Regimen 10 (TDF + 3TC + LPV/r)
        12. Regimen 11 (AZT + 3TC + LTV/r)
        13. Regimen 12 (DRV + r + ETV + RAL)
        14. Other
    35. Since we recruited participant to this study (Day 0: ____) and today, was participant admitted to hospital for any reason? By "admitted to hospital", I mean stayed at least one night in hospital *
        1. Yes
        2. No
    36. Since we recruited participant to this study (Day 0: ____) and today, how many times was participant admitted to hospital? By this, I mean how many episodes did participant have when they stayed in hospital for at least one night. *
    37. Hospital admission episode number
    38. You have told me that the participant was admitted to hospital a total of ____ times. I am now going to ask you about episode number: ____
    39. On what date was participant admitted to hospital?
    40. During this admission, how many nights did participant spend in hospital in total? *
    41. During this admission, what was the reason for participant's admission? *
        1. Diarrhoea (sudden, or with blood)
        2. Anaemia
        3. Thrush in the mouth or throat
        4. Stroke
        5. Diarrhoea (that has gone on for a long time)
        6. Heart failure
        7. Cryptococcal meningitis
        8. Diabetes (with complications)
        9. Diabetes (without complications)
        10. Diseases of the kidneys, bladder or womb
        11. Muscle or bone problems
        12. Epilepsy / seizures / convulsion
        13. High blood pressure (hypertension)
        14. Kaposi's sarcoma
        15. Liver disease
        16. Malaria
        17. Meningitis
        18. Mental health problems
        19. Cancers (excluding Kaposi's sarcoma)
        20. Other heart (cardiovascular problems)
        21. Other brain or nerve (neurological) problems
        22. Other breathing/lung problems
        23. Pneumocystis Jivorecii Pneumonia (PCP)
        24. Pneumonia (infection of the lungs)
        25. Pregnancy / maternity related
        26. TB of the lungs (pulmonary TB)
        27. Blood infection (scepticaemia)
        28. Tuberculosis (retreatment)
        29. Tuberculosis of bones and joints
        30. Tuberculosis of the gut/stomach
        31. Tuberculosis of the brain / meningitis
        32. Miliary tuberculosis (TB throughout the whole body)
        33. Tuberculosis of any other organ
        34. Upper gastrointestinal problems (problems with the throat or stomach)
        35. Viral infection
        36. Any other reason
        37. Don't know/can't remember
    42. During this admission, did participant have any sputum tests done for TB testing? (check all that apply) *
        1. Sputum smear
        2. GeneXpert test
        3. Sputum culture
        4. No sputum tests done
        5. Don't know
    43. During this admission, did participant have any x-ray tests done? *
        1. Chest x-ray done
        2. Another x-ray done
        3. No x-rays done
        4. Don't know
    44. During this admission, did participant start treatment for tuberculosis? *
        1. Yes
        2. No
        3. Don't know/can't remember
    45. During this admission, did participant have an HIV test done? *
        1. Yes
        2. No
        3. Don't know/can't remember
    46. During this admission, did participant start ART treatment for HIV? *
        1. Yes
        2. No
        3. Don't know/can't remember
    47. During this admission, did participant have any blood tests done? *
        1. Yes
        2. No
        3. Don't know/can't remember
    48. During this admission, did participant have any antibiotic injections? *
        1. Yes
        2. No
        3. Don't know/can't remember
    49. For how many days did participant receive antibiotic injections?
    50. Since we recruited participant to this study (Day 0: ____), and today, has particiapnt attended any health centres or clinics? *
        1. Yes
        2. No
    51. Since we recruited the participant to this study (Day 0: ____), and today, how many times has the participant attended a health centre or clinic? *
    52. Clinic attendance episode number
    53. You have told me that the participant attended a clinic or health centre total of ____ times. I am now going to ask you about clinic attendance episode number: ____
    54. On what day did this clinic attendance happen? *
    55. On this clinic attendance, which clinic did participant attend? *
        1. Bangwe (BG)
        2. BTGW
        3. Chilomoni (CH)
        4. Chitawira (CW)
        5. Gateway Clinic (GW)
        6. Limbe Health Centre (LB)
        7. Mlambe (MB)
        8. Mwaiwathu (MW)
        9. Ndirande (ND)
        10. Queen Elizabeth Central Hospital (QE)
        11. South Lunzu (SL)
        12. Zingwangwa (ZW)
        13. Other
    56. What was participant's main reason for attending the clinic on this occasion? *
        1. For HIV testing/counselling
        2. To collect antiretroviral therapy for treating HIV
        3. To attend HIV care clinic (not taking antiretroviral therapy)
        4. To register for, or collect, TB treatment
        5. Hypertension/high blood pressure clinic (routine follow-up)
        6. Diabetes clinic (routine follow-up)
        7. Antenatal clinic / maternity clinic
        8. Feeling unwell / wanted to get treatment
        9. Other reason
        10. Don't know
    57. During this clinic attendance, did participant have any sputum tests done for TB testing? (check all that apply) *
        1. Sputum smear
        2. GeneXpert test
        3. Sputum culture
        4. No sputum tests done
        5. Don't know
    58. During this attendace, did participant have any x-ray tests done? *
        1. Chest x-ray done
        2. Another x-ray done
        3. No x-rays done
        4. Don't know
    59. During this attendance, did participant start treatment for tuberculosis? *
        1. Yes
        2. No
        3. Don't know/can't remember
    60. During this admission, did participant have an HIV test done? *
        1. Yes
        2. No
        3. Don't know
    61. During this attendance, did participant start ART treatment for HIV? *
        1. Yes
        2. No
        3. Don't know/can't remember
    62. During this attendance, did participant have any blood tests done? *
        1. Yes
        2. No
        3. Don't know
    63. During this attendance, did participant receive any antibiotic tablets? *
        1. Yes
        2. No
        3. Don't know/can't remember
    64. For how many days did the health worker tell the participant to take the antibiotic tablets?
    65. How do you rate your general health? *
        1. Very good
        2. Good
        3. Fair
        4. Poor
        5. Very poor
    66. Mobility: Which statements best describe your own health state today? *
        1. I have no problems in walking about
        2. I have some problems in walking about
        3. I am confined to bed
    67. Self-care: Which statements best describe your own health state today? *
        1. I have no problems with self-care
        2. I have some problems washing or dressing myself
        3. I am unable to wash or dress myself
    68. Usual Activities (e.g. work, study, housework, family or leisure activities): Which statement best describe your own health state today? *
        1. I have no problems with performing my usual activities
        2. I have some problems with performing my usual activities
        3. I am unable to perform my usual activities
    69. Pain/Discomfort: Which statements best describe your own health state today? *
        1. I have no pain or discomfort
        2. I have moderate pain or discomfort
        3. I have extreme pain or discomfort
    70. Anxiety/Depression: Which statements best describe your own health state today? *
        1. I am not anxious or depressed
        2. I am moderately anxious or depressed
        3. I am extremely anxious or depressed
    71. To help people say how good or bad a health state is, we have drawn a scale (rather like a thermometer) on which the best state you can imagine is marked 100 and the worst state you can imagine is marked 0. We would like you to indicate on this scale how good or bad your own health is today, in your opinion. Please do this by drawing a line from the box below to whichever point on the scale indicates how good or bad your health state is today. *
    72. Height (in cm) *
    73. Weight (in kg) *
    74. Does participant still have sputum cups and laboratory request form with barcode stickers attached? *
        1. Yes
        2. No
    75. Attach new barcodes to new sputum cups and laboratory form, and scan new barcode *
    76. Did participant successfully provide 2x sputum samples *
        1. Yes
        2. No
    77. Inform participant that they will only be contacted if their TB sputum test result is positive. Thank them for participating in the study.
    78. * Ask participants to try producing sputum again tomorrow morning. * Arrange a follow-up home visit tomorrow to collect samples. * Record in Study Register that sputum samples have not yet been collected.
    79. Now complete FORM E (HIV Testing Episode) and FORM H (Day 56 TB Screening Episode)
    80. #form/j9999_complete
    81. #form/j0152_attendno/j01511_hidden
    82. #form/j0140_episode/j01401_hidden

##### [K HIV care clinic registration](file:////a/prospect-1/apps/view/2a235d4096443d62135df383d58bb999/form/88f4609e7df59664768de92aaa87151f66a5de5/)

- - 1. Use this form to record a participant's registration for HIV care and ART assessment as a result of study interventions
    2. Participant registered at HIV care clinic *
       1. Yes
       2. No
    3. HIV care clinic number *
    4. Baseline WHO clinical Stage *
       1. Stage 1
       2. Stage 2
       3. Stage 3
       4. Stage 4
       5. Not Staged
    5. ART initiated? *
       1. Yes
       2. No
    6. Date ART initiated *
    7. Which ART medication regimen was initiated? *
       1. Regimen 0 (ABC + 3TC + NVP)
       2. Regimen 1 (D4T + 3TC + NVP)
       3. Regimen 2 (AZT + 3TC + NVP)
       4. Regimen 3 (D4T + 3TC + EFV)
       5. Regimen 4 (AZT + 3TC + EFV)
       6. Regimen 5 (TDF + 3TC + EFV)
       7. Regimen 6 (TDF + 3TC + NVP)
       8. Regimen 7 (TDF + 3TC + ATV/r)
       9. Regimen 8 (AZT + 3TC + ATV/r)
       10. Regimen 9 (ABC + 3TC + LPV/r)
       11. Regimen 10 (TDF + 3TC + LPV/r)
       12. Regimen 11 (AZT + 3TC + LTV/r)
       13. Regimen 12 (DRV + r + ETV + RAL)
       14. Other
    8. #form/k9999_complete

##### [L TB registration](file:////a/prospect-1/apps/view/2a235d4096443d62135df383d58bb999/form/7cb9dcc01f605176dd87788d0a7c44474864504b/)

- - 1. Use this form to record a participant's registration forTB treatment as a result of study interventions
    2. Which TB treatment centre did participant register for treatment at? *
       1. Blantyre Adventist Hospital (BAH)
       2. Bangwe (BG)
       3. BTGW
       4. Chilomoni (CH)
       5. Chitawira (CW)
       6. Gateway Clinic (GW)
       7. Limbe Health Centre (LB)
       8. Mlambe (MB)
       9. Mwaiwathu (MW)
       10. Ndirande (ND)
       11. Queen Elizabeth Central Hospital (QE)
       12. Queen Elizabeth Central Hospital 2 (QE2)
       13. South Lunzu (SL)
       14. Zingwangwa (ZW)
       15. Other
    3. TB Registration number *
    4. Date of TB registration *
    5. Date TB treatment started *
    6. Tuberculosis disease site (check one) *
       1. Pulmonary
       2. Extra-Pulmonary
    7. Sputum smear result *
       1. Sputum smear positive
       2. Sputum smear negative
       3. Not done
    8. Xpert MTB/Rif results *
       1. MTB detected
       2. MTB not detected
       3. Not done/laboratory problem
    9. Xpert MTB/Rif resistance testing result *
       1. RIF resistance detected
       2. RIF Resistance not detected
       3. Not done/laboratory problem
    10. Chest x-ray result *
        1. Chest x-ray suggestive of TB
        2. CXR not suggestive of TB
        3. Not done
    11. TB culture result *
        1. TB culture positive
        2. Culture negative
        3. Not done
    12. Type of TB patient (check one) *
        1. New
        2. Relapse
        3. Transfer in
        4. Treatment after default
        5. Treatment after failure
        6. Other
    13. Initial TB treatment phase drugs (select all that apply) *
        1. RHZE
        2. S
        3. Other
    14. Continuation Phase TB treatment drugs (select all that apply) *
        1. RH
        2. RHE
        3. Other
        4. Don't know
    15. #form/l9999_complete

##### [M Adverse event](file:////a/prospect-1/apps/view/2a235d4096443d62135df383d58bb999/form/2c09beec230722d4f2f613f63af4748659fa035a/)

- - 1. Who was interviewed? *
       1. Participant
       2. Another household member
       3. Someone else
    2. Is participant deceased? *
       1. Yes
       2. No
    3. Date of death *
    4. Was participant taking TB treatment in period before death? *
       1. Yes
       2. No
       3. Don't know
    5. Date TB treatment started *
    6. Was participant known to be HIV-positive? *
       1. Yes
       2. No
       3. Don't know
    7. Was participant taking ART in the period before death? *
       1. Yes
       2. No
    8. Date that participant started ART *
    9. Date that event happened *
    10. Time that event happened *
        1. Morning
        2. Afternoon
        3. Evening
        4. Night
    11. Status of event *
        1. New
        2. Ongoing
    12. Location of event *
        1. Home
        2. In the community
        3. Research office
        4. Hospital or clinic
        5. Somewhere else
    13. Who was involved in the event? *
        1. Participant
        2. Household/family member
        3. Another study participant
        4. Another patient
        5. Another community membe
        6. Study team member
        7. Clinic/hospital staff
    14. Type of event *
        1. Misclassification of HIV result
        2. Misclassification of TB results
        3. Breach of confidentiality
        4. Other (describe)
        5. Needlestick injury
    15. Give a detailed description of the event *
    16. What type of untoward event is this? *
        1. Adverse event
        2. Adverse reaction
        3. Unexpected adverse reaction
        4. Serious adverse event
        5. Serious adverse reaction
        6. Suspected unexpected serious adverse reaction
    17. Has the study PI been informed about this event? *
        1. Yes
        2. No
    18. Describe action taken in relation to this event *
    19. Is the event related to study interventions? *
        1. Yes
        2. No
    20. Outcome *
        1. Resolved
        2. Ongoing

##### [N Study termination](file:////a/prospect-1/apps/view/2a235d4096443d62135df383d58bb999/form/1af741495b5db47997112da3787bfdc864d8a83b/)

- - 1. This form should be completed for all participants that have been terminated from the study.
    2. Confirm this participant has withdrawn from the PROSPECT Study *
       1. I confirm participant has been withdrawn from study (select)
    3. Date of termination from study *
    4. Reason participant terminated from study *
       1. Death (participant died during study period up to day 56)
       2. Lost to follow-up
       3. Relocated
       4. Following PI's decision
       5. Withdrew consent
       6. Other
       7. Withdrew consent for fingerprinting
    5. Please provide a description of the reason for termination from study. Include events leading up to termination, and any action taken. *
    6. #form/n9999_complete

##### [Q Exit interview (Quality assurance)](file:////a/prospect-1/apps/view/2a235d4096443d62135df383d58bb999/form/5f19eb2342d9c7b7ea749c446a2a5f42adf30fa/)

- - 1. If person is not recruited as participant in the PROSPECT Study trial, please complete a consent form, explaining about the process of the exit interview.
    2. Have you ever taken treatment for tuberculosis previously? *
       1. Yes
       2. No
    3. Have you ever been tested for HIV? *
       1. Yes
       2. No
    4. In the last 12 months, how many times have you tested for HIV *
    5. You don't have to tell me if you don't want, but what was the result of your last HIV test? *
       1. Positive
       2. Negative
       3. Don't know
       4. Don't want to reveal
       5. Never tested
    6. Have you ever had a positive HIV test? *
       1. Yes
       2. No
    7. Are you currently taking ART drugs? *
       1. Yes
       2. No
    8. Now I want to ask you some questions about what happened during your visit to the clinic today
    9. Were you seen by a clinic health worker today? *
       1. Yes
       2. No
    10. Did the health worker ask you if you had a cough today? *
        1. Yes
        2. No
    11. Were you asked to give a sputum sample? *
        1. Yes
        2. No
    12. Did you give a sample of sputum to the health worker today?
        1. Yes
        2. No
    13. Did you get the results of the sputum test today?
        1. Yes
        2. No
    14. Were you told the result of the sputum test today?
        1. Yes
        2. No
    15. Did you have an x-ray of your chest in the clinic today? *
        1. Yes
        2. No
    16. Were you told that you had tuberculosis (TB) today? *
        1. Yes
        2. No
    17. Were you told that you needed to start treatment for TB today? *
        1. Yes
        2. No
    18. Did you start treatment for TB today? *
        1. Yes
        2. No
    19. Were you offered a test for HIV today? *
        1. Yes
        2. No
    20. Did you do a test for HIV today? *
        1. Yes
        2. No
    21. Were you told the result of your HIV test today? *
        1. Yes
        2. No
    22. Were you told you needed to start treatment for HIV? *
        1. Yes
        2. No
    23. Did you start treatment for HIV (ART) today? *
        1. Yes
        2. No
    24. How do you rate your general health? *
        1. Very good
        2. Good
        3. Fair
        4. Poor
        5. Very poor
    25. Thinking about your experience in the clinic today, would would you say it has been... *
        1. Excellent
        2. Good
        3. Fair
        4. Poor
    26. Did you bring your health passport today? *
        1. Yes
        2. No
    27. Why didn't you bring your health passport today? *
        1. Forgot to bring it
        2. Lost it
        3. It's damaged
        4. I didn't know I needed it
    28. Have you ever lent out your health passport? *
        1. Yes
        2. No

##### [R Re-attendance](file:////a/prospect-1/apps/view/2a235d4096443d62135df383d58bb999/form/a83e92521d1995a4d926b4e2fd116f9f531fe428/)

- - 1. This form should be completed for individuals who were registered into the PROSPECT Study, and then reattend the clinic.
    2. This study participant should be assessed in the Study Room for day 56 assessment. Please take them to the study room.
    3. This individual has not been recruited to the PROSPECT Study. Please re-assess their eligibility by completing Form A Eligibility and Consent
    4. This individual is a participant in the PROSPECT Study, but their Day 56 appointment is not yet due. Please refer them to the clinic waiting area. Remind them of the date and time of their Day 56 appointment

##### [S Update Phone Number](file:////a/prospect-1/apps/view/2a235d4096443d62135df383d58bb999/form/fbc1e067c5ff77ff1f20ebdc5ef418468776cd66/)

- - 1. Phone Number
