# Supplementary material for: Design and protocol for a pragmatic randomised study to optimise screening, prevention and care for tuberculosis and HIV in Malawi (PROSPECT Study)
Source: Wellcome Open Res. 2018 Nov 21;3:61. Originally published 2018 May 18. [Version 3] doi: 10.12688/wellcomeopenres.14598.3 (PMC6259593; doi:10.12688/wellcomeopenres.14598.3)
Supplement: Peer review history

# Peer review: Referee response for version 3

**DOI:** 10.21956/wellcomeopenres.16287.r34293

**Published:** 2018-11-26

**Authors:** Tom A. Yates

**Affiliations:** 1 Section of Infectious Diseases and Immunity, Imperial College London, London, UK

Apologies for my delay in responding to these revisions. It is important that protocols are published before trials are undertaken and this document should now be indexed on databases. A few final thoughts below.

 First, the authors are to be congratulated on undertaking a randomised evaluation of a carefully considered intervention with the potential to make a significant impact on avoidable morbidity and mortality. Robust evaluations of such programatic interventions (which usually means randomisation) are both challenging and incredibly important.

 Thought will need to be exercised in interpreting the results of this trial, when it reports.

 In the event of a null result, the possibility that the trial intervention has improved outcomes in the standard of care arm will need to be considered. The authors should think now about whether there are process data that might be collected to help understand a null result. Sophisticated analyses of null trial results can be instructive – for example, the work that was done to explain the results of the Thibela trial.

 I agree that the proportion of participants at day 56 with undiagnosed microbiologically confirmed TB is a key outcome. I also agree that, in this context, under treatment is far riskier than over treatment. I still think that the trial would be stronger were baseline sputum samples collected from (at least) those initiating treatment at the initial study visit. This might be done, without introducing bias, at an exit interview. I appreciate that there are considerable resource implications but these data would assist in understanding the extent of overtreatment, an outcome of considerable importance for patients.

 Anyway, enough from me. Good luck with this important study. I very much look forward to seeing the results.

I have read this submission. I believe that I have an appropriate level of expertise to confirm that it is of an acceptable scientific standard.

# Peer review: Referee response for version 2

**DOI:** 10.21956/wellcomeopenres.16035.r33594

**Published:** 2018-10-02

**Authors:** Frank Cobelens

**Affiliations:** 1 Department of Global Health, Academic Medical Center, Amsterdam, The Netherlands; 2 Amsterdam Institute for Global Health and Development, Amsterdam, The Netherlands

I thank the authors for their responses to the comments and revisions to the manuscript. I find all comments well addressed and approve the present version for indexing.

I have read this submission. I believe that I have an appropriate level of expertise to confirm that it is of an acceptable scientific standard.

# Peer review: Referee response for version 1

**DOI:** 10.21956/wellcomeopenres.15894.r33288

**Published:** 2018-06-22

**Authors:** Tom A. Yates

**Affiliations:** 1 Section of Infectious Diseases and Immunity, Imperial College London, London, UK

Thanks for asking me to review this protocol. I have read the protocol and the thoughtful peer review by Frank Cobelens. I have not had an opportunity to read the supplementary material. I comment as a clinician with training in epidemiology. The editors may wish to request a statistical review and the views of a health economist.

 I have divided my comments into those focused on the trial design and those focused on the description of the proposed study in the protocol.

**Comments on the design of the trial**

- I share Frank’s concerns about the potential for the trial interventions to alter clinical practice in the standard of care arm. The authors acknowledge this possibility and state that, should the intervention appear effective in this trial, they might proceed to a cluster RCT, randomising clinics. I would note that the Hawthorne Effect here would be more likely to bias the effect estimate towards the null, rather than lead to a spurious positive result.
- I think Frank’s comment about the primary outcome measure not capturing the number of people initiating TB treatment is an important one. I would add that, ideally, the primary outcome measure should consider whether initiation of TB treatment was appropriate or not. Empirical TB treatment may be more common in the standard of care arm, where clinicians may have less ready access to investigations. Early initiation of TB treatment in people who do not have TB is not a good thing. I would like to see more discussion about this possibility.
- Not having cultures from all participants at baseline will make interpretation of the results difficult, particularly if there is a lot of empirical TB treatment (as I expect there may be). A good number of these patients may not have TB and unpicking that at day 56, by which time true cases of TB may have culture converted, may not be possible. Possible approaches to dealing with this issue…
  a) Take sputum for culture from everyone at baseline but don’t feed the result back to clinicians until day 56. Is this morally less acceptable than deliberately not obtaining a sputum specimen? I would point out that delaying sputum collection until day 56 would lead to treatment initiation being further delayed in patients whose smear negative/Xpert negative TB had not already been diagnosed.
  b) Request that a baseline sputum sample for culture be taken from everyone in whom a decision to initiate TB treatment is made.
- It would be good to see much more detail about what will happen during the day 56 study visit. If systematic recording of the adverse outcomes is to be achieved (especially ‘Misclassification or misinterpretation of results leading to a participant starting TB therapy in error’), then presumably some determinantion will need to be made about which patients truly have TB and which do not. More detail about how this will be done should be included in the protocol, including how it will be done in patients that die before day 56.
- It seems likely that clinicians making this assessment will need to speak to the patient (perhaps their relatives or clinicians who looked after them in hospital where patients have died) and to consult clinic notes from the first study visit. I struggle to see how this can be done whilst blinding the clinician to the group to which the patient was randomised, as is suggested will happen in the protocol.
- I would suggest tempering the comments about this study being pragmatic. It strikes me that the trial is somewhere on the continuum between an efficacy trial and a truly pragmatic trial. Were this a truly pragmatic trial, the trial interventions would be implemented by existing clinic staff. Here, study staff implement the interventions. I would like the authors to comment on how they will unpick the impact of the trial interventions from the impact of the additional human resources available to patients randomised to Groups 2 and 3. It seems possible (even likely) that the trial interventions would be less effective if they were being implemented by busy clinic nurses?
- I am not a health economist but wonder whether any differences in EQ5D scores might be diluted by including in this comparison lots of people who don’t have TB (in whom the impact of the intervention may be minimal).
- Frank’s comment about non TB findings on chest x-ray is an important one. Might this trial be a good opportunity to quantify the burden of non TB disease that might be missed and whether that varies depending on whether clinicians have access to CAD?
- With regards the second secondary outcome, I am unclear as to the rationale for excluding people that have stopped TB treatment for good reason (e.g. hepatotoxicity)?
- With regards the nested evaluation of the CAD4TB tool, gold standard definitions of TB have been defined. Do gold standard definitions of ‘not TB’ need to also be defined, to allow estimates of specificity? I am not clear what is meant by ‘reader characteristics’ in the final paragraph of this section.
- People with previous TB make up a significant proportion of those with TB disease in high burden settings (Florian Marx has done some nice work describing this phenomenon). These people may have residual x-ray changes and there are reports of false positive Xpert tests in individuals who have been recently treated. Will excluding people that have had TB within the preceding six months deal with the latter issue, or can DNA persist for longer than this in people with recent successfully treated TB?
- I would like some justification for the Bayesian analysis to be included in the protocol. What is this achieving that couldn’t be achieved using a frequentist approach?
- I really like the open approach to sharing data and code that is proposed by the investigators.

 

**Comments on the trial protocol**

- It seems like enrollment will precede randomisation but I would like to see a more explicit comment on allocation concealment.
- I would like to see more comment about how patients who transfer their care out or are lost to follow up will be handled in the analysis.
- The CAD threshold that will be used should be pre-specified in the protocol.
- A note should be made on the approach that will be taken to x-rays in women of childbearing age.
- A note should be made about the approach that will be taken to patients that have RpoB mutations detected by Xpert.
- It seems unlikely that the trial team will be able to see all participants on day 56. I suggest that the team allow themselves a window of time in which to undertake these study visits.
- Please include an explicit comment about whether/how the urine LAM result will be fed back to clinicians.
- There are a number of grammatical errors and the writing, in places, could be clearer. The section on trial outcomes, in particular, could be better written.

 

**Conclusions**

 The proposed two step approach to evaluating people with suspected pulmonary TB in settings such as these seems to be a good idea. It is good to see this proposal to evaluate the approach in a randomised controlled trial. My main concerns about the protocol concern the choice of primary outcome, particularly how this might perform in a setting where clinicians often initiate empirical TB treatment. An attempt to ascertain ‘true TB status’ would be valuable in interpreting the findings of the trial. The approach that will be taken to define ‘true TB status’ should be described in greater detail. I suggest, as a minimum, that an attempt be made to obtain a baseline sputum sample for TB culture in all those starting TB treatment, regardless of study arm.

I have read this submission. I believe that I have an appropriate level of expertise to confirm that it is of an acceptable scientific standard, however I have significant reservations, as outlined above.

# Response

**Published:** 2018-07-23

**Authors:** Peter MacPherson

**Affiliations:** Liverpool School of Tropical Medicine, Malawi

We are grateful for the detailed and helpful reviews provided by Tom Yates. We have responded in detail to all comments below.

**1.** **I share Frank’s concerns about the potential for the trial interventions to alter clinical practice in the standard of care arm. The authors acknowledge this possibility and state that, should the intervention appear effective in this trial, they might proceed to a cluster RCT, randomising clinics. I would note that the Hawthorne Effect here would be more likely to bias the effect estimate towards the null, rather than lead to a spurious positive result.**

Thank you – we considered a number of different study designs, including a clinic cluster-randomised trial (would require an extremely large budget and massively increased resources), to an observational before-and-after design (with concomitant greater concerns around bias). No study design is perfect, but we hope that the single clinic pragmatic trial design we selected will allow us to provide strong, policy-relevant evidence. Should interventions be cost-effective in this trial, there would be a compelling argument for a subsequent cluster-randomised trial, or implementation evaluation as part of scale-up. We believe that any Hawthorne effect wold be more likely to bias effect estimate towards the null.

**2.** **I think Frank’s comment about the primary outcome measure not capturing the number of people initiating TB treatment is an important one. I would add that, ideally, the primary outcome measure should consider whether initiation of TB treatment was appropriate or not. Empirical TB treatment may be more common in the standard of care arm, where clinicians may have less ready access to investigations. Early initiation of TB treatment in people who do not have TB is not a good thing. I would like to see more discussion about this possibility.**

We agree this is a difficult area. Right now, rates of empirical treatment are high, but exit interviews and high rate of prevalent TB (particularly among ART initiators) suggest that still far too few people with TB symptoms are evaluated appropriately, diagnosed, and linked promptly to treatment. We suspect that there is substantial under-diagnosis of TB among clinic attenders with current symptom-screen/sputum-based diagnostic approaches. We agree that clearly understanding the proportion of TB patient who are correctly started on TB would be of benefit. However, we believe that there is potentially greater public health benefit to be obtained by optimising available screening tests and linkage systems, and (hopefully) reducing time to treatment. We will be able to evaluate this to some extent by examining the proportion of participants with undiagnosed microbiologically-confirmed TB at Day 56.

Mathematical modelling studies suggest that an algorithm comprised of an initial chest x-ray, followed by confirmatory GeneXpert is likely to have achieve high sensitivity and specificity at acceptable programme costs. As discussed in detail in response to Point 3 below, the considerable additional resources required to ascertain microbiological TB status among all participants, coupled with the potential to compromise the study validity and strength of the evidence available from the trial, means that this approach is not feasible.

**3.** **Not having cultures from all participants at baseline will make interpretation of the results difficult, particularly if there is a lot of empirical TB treatment (as I expect there may be). A good number of these patients may not have TB and unpicking that at day 56, by which time true cases of TB may have culture converted, may not be possible. Possible approaches to dealing with this issue…**

**a) Take sputum for culture from everyone at baseline but don’t feed the result back to clinicians until day 56. Is this morally less acceptable than deliberately not obtaining a sputum specimen? I would point out that delaying sputum collection until day 56 would lead to treatment initiation being further delayed in patients whose smear negative/Xpert negative TB had not already been diagnosed.**

**b) Request that a baseline sputum sample for culture be taken from everyone in whom a decision to initiate TB treatment is made.**

Thank you for raising these important issues. The trial team considered this in some detail. Given that TB culture is not routinely available in Malawi and most other countries in sub-Saharan Africa, we felt that doing sputum culture for TB for all participants at baseline and providing results to participants would not reflect current standard of care, and would provide evidence that was of lesser direct relevance to policymakers. We note that in the TBNeat Trial (Theron et al, Lancet 2014), although rates of empirical TB treatment were high, study TB culture taken at baseline and reported back to clinic nurses did provide additionality in TB treatment initiations; in the Smear Microscopy and Xpert Arms respectively, 18/154 (12%) and 15/170 (9%) of TB treatment initiations were attributed to a positive TB culture result. In Malawi under current standard of care, these patients would not achieve a microbiologically-confirmed diagnosis. It is possible that a baseline culture in our setting might attenuate differences in TB treatment initiations between groups, whilst diminishing the relevance of evidence we are able to provide to policymakers in the region.

We didn’t feel it would be ethically acceptable to collect sputum and perform TB culture, but not report results to patients or clinicians, especially given the precedence already set in studies such as Theron et al.

We did carefully consider doing TB cultures for participants initiating TB treatment. However, there are a number of constraints to this. TB treatment will not be initiated by study Research Assistants, but by routine facility staff. In Groups 1 and 2, Study Research Assistants will not be present when TB treatment is initiated; all decisions to initiate TB treatment and registration procedures will be done by routine clinic healthworkers. In Group 3, participants may be linked to TB registration by study Research Assistants; however, it is possible that healthworkers will make a clinical decision to initiate TB treatment between Day 0 and Day 56 for participants not linked to registration by study team members at baseline. In such a case, there would be no Research Assistants present to collect sputum. Additionally, in all three groups, participants may initiate TB treatment at another clinic or hospital in Blantyre during the follow-up period (for example, they may be admitted to the central hospital, and registered for TB treatment there by routine healthworkers). 

As part of a separate cluster-randomised trial (the HitTB Study), we attempted to collect a single spot sputum sample from all patients registering for TB treatment at all 18 health facilities in Blantyre. Citywide, we successfully collect sputum from 73% of all registering TB cases, demonstrating the challenges of achieving high levels of case ascertainment under routine programmatic conditions.

Overall, we remain unconvinced that attempting to collect a sputum sample for culture from all participants registering for TB treatment would be feasible, affordable, or sufficiently free of bias. Indeed, we suspect that there would be likely substantially greater ascertainment of TB culture status among participants in Group 3 than from the other two groups, hindering our ability to draw meaningful conclusions.

**4.** **It would be good to see much more detail about what will happen during the day 56 study visit. If systematic recording of the adverse outcomes is to be achieved (especially ‘Misclassification or misinterpretation of results leading to a participant starting TB therapy in error’), then presumably some determinantion will need to be made about which patients truly have TB and which do not. More detail about how this will be done should be included in the protocol, including how it will be done in patients that die before day 56.**

We described the procedures undertaken at Day 56 as follows:

*“Following completion of trial interventions, all participants in each of the three groups will be given a written appointment card to attend a follow-up assessment at the study research clinic room 56 days after randomisation. They will also be issued with a voucher that they can use to reimburse the cost of transport to the clinic for this assessment. Participants who don’t attend their day 56 appointment will be traced to home.*

*To evaluate the primary trial outcome (time to tuberculosis treatment initiation), we will undertake a detailed questionnaire to record the date of TB treatment initiation, and verify by inspecting participant-carried TB treatment cards, medication and clinic TB treatment registers.*

*To evaluate the prevalence of undiagnosed tuberculosis, we will collect sputa from all participants. Samples will be transported to the TB Research Laboratory at the College of Medicine of Malawi, where they will be cultured for tuberculosis using the MGIT system, undergo smear examination using fluorescence microscopy, and tested using the GeneXpert MTB/Rif assay. Positive TB results will be reported to participants within three days of receipt (including by home tracing), and participants will be supported to register for TB treatment at the health facility.*

*To evaluate the prevalence of undiagnosed HIV at Day 56, we will offer all participants HIV testing, unless they are confirmed to be taking antiretroviral therapy. All participants requiring additional care will be supported to access either the HIV clinic, TB clinic, or outpatient clinic at the study clinic as required. Additionally, we will support referral and access to Queen Elizabeth Central Hospital should further specialist care be required.*

*To evaluate health-related quality of life (HRQoL), we will use the EQ-5D-3L. The EQ-5D is a generic HRQoL measure, and was translated into Chichewa following international and EuroQoL guidelines. The EQ-5D-3L tool will be administered to all trial participants at baseline, and on Day 56.”*

We respectfully disagree that outcome assessment requires a definitive classification of whether a participant truly had TB or not. As discussed in response to Point 3 above, our trial primary outcome sets out to investigate time to TB treatment initiation among all participants, reflecting the reality of how TB care is currently delivered in low-resource settings. Secondary outcomes will evaluate the prevalence of undiagnosed TB, and proportion of participants achieving same-day treatment of TB. We don’t think it is feasible to obtain a definitive microbiological TB status on all participants, as this would compromise our ability to provide unbiased evidence that is comparable to how diagnosis and care are currently delivered in Malawi and other countries in the region.

Relating to the adverse outcome of misclassification of results, we will record and report instances where a study-conducted GeneXpert is mistakenly reported to a participant as positive, when in fact results indicate it was negative (or vice-versa); or where a study-conducted culture is either mistakenly reported to a participant as positive, or whether misinterpretation leads to a delay in reporting results to a participant.

All participants who don’t attend their day 56 outcome assessment will be traced to home, and their household members interviewed to ascertain TB and HIV treatment status in the period prior to death. We will not be able to take sputum samples from participants who have died before Day 56, so these secondary outcomes will be missing. In the preplanned sensitivity analysis (see the Statistical Analysis Plan in the Supplemental Material) we describe how this will be addressed:

*“Participants who have missing information for outcomes will be excluded from primary analysis. However, in sensitivity analysis, we will use multiple imputation by chained equations to replace missing outcome variables.”*

**5.** **It seems likely that clinicians making this assessment will need to speak to the patient (perhaps their relatives or clinicians who looked after them in hospital where patients have died) and to consult clinic notes from the first study visit. I struggle to see how this can be done whilst blinding the clinician to the group to which the patient was randomised, as is suggested will happen in the protocol.**

The protocol describes procedures for ascertaining TB treatment initiation status at the Day 56 outcome visit:

*“To evaluate the primary trial outcome (time to tuberculosis treatment initiation), Research Assistants will undertake a detailed questionnaire to record the date of TB treatment initiation, and verify by inspecting participant-carried TB treatment cards, medication and clinic TB treatment registers.”*

No clinicians will undertake outcome assessments; these will be done by study Research Assistants. In the Outcome Assessment section, we have replaced the word “we” with “Research Assistants” to add clarity. All participants (regardless of group) will receive the same procedures at Day 56 assessment, meaning that the likelihood of differential outcome assessment is reduced. We will only interview caregivers during home tracing for participants who have died.

**6.** **I would suggest tempering the comments about this study being pragmatic. It strikes me that the trial is somewhere on the continuum between an efficacy trial and a truly pragmatic trial. Were this a truly pragmatic trial, the trial interventions would be implemented by existing clinic staff. Here, study staff implement the interventions. I would like the authors to comment on how they will unpick the impact of the trial interventions from the impact of the additional human resources available to patients randomised to Groups 2 and 3. It seems possible (even likely) that the trial interventions would be less effective if they were being implemented by busy clinic nurses?**

We agree that the definition of a pragmatic trial exists on a spectrum. Having interventions delivered by routine clinic staff is not the sole criteria that defines a pragmatic trial. Important other considerations include: whether a single intervention, or multiple complex interventions are evaluated; whether patient-focused/important measures (as opposed to biological parameters) are used as outcomes; whether routine health system data are used to assess outcomes; whether interventions are integrated within routine healthcare delivery. These issues and definitions are reviewed in Sox et al JAMA 2016. In this study, interventions (TB testing, HIV testing, linkage to treatment, other care services) may be also be provided through the routine clinic system by routine healthworkers in all three trial Groups, as shown in Figure 3. We feel that the design of the study places it firmly towards the pragmatic end of the spectrum.

Note, when we specify “clinicians” we mean non-physician healthworker cadres (Clinical Officers, Nurses), who provide much of the care in primary health care clinics. In the study clinic, there are no physicians. This reflects how care is delivered in primary clinics in much of sub-Saharan Africa. We hope that by conducting the study in a typical primary health care clinic, we will be able to provide evidence for cost-effectiveness under “real-life” conditions.

**7.** **I am not a health economist but wonder whether any differences in EQ5D scores might be diluted by including in this comparison lots of people who don’t have TB (in whom the impact of the intervention may be minimal).**

We expect that, if random allocation is successful, the proportion of participants with TB in each arm will be similar. This outcome will evaluate the effect of interventions on change in EQ5D score among adults attending primary care with symptoms of TB. The Reviewer is correct that there is likely to be a wider range of EQ5D scores at baseline than if we included only patients with “true” TB, however, we don’t think this would be possible for reasons described above in response to Point 3. We believe, and ours and others studies show, that adults attending primary health care with symptoms of TB often experience poor quality of care, and only a small fraction are investigated appropriately for both TB and HIV. Here, we hypothesise that optimised screening and linkage to care interventions for TB and HIV could improve the quality of life for adults with symptoms of TB.

**8. Frank’s comment about non TB findings on chest x-ray is an important one. Might this trial be a good opportunity to quantify the burden of non TB disease that might be missed and whether that varies depending on whether clinicians have access to CAD?** 

This is a good suggestion for additional exploratory analysis. Thank you.

**9.** **With regards the second secondary outcome, I am unclear as to the rationale for excluding people that have stopped TB treatment for good reason (e.g. hepatotoxicity)?**

Thanks – this was purely a pragmatic decision. Usually it is not possible to determine reason for TB treatment being stopped from TB treatment cards or registers. Therefore, we took the decision to exclude all such cases from the numerator.

**10.** **With regards the nested evaluation of the CAD4TB tool, gold standard definitions of TB have been defined. Do gold standard definitions of ‘not TB’ need to also be defined, to allow estimates of specificity? I am not clear what is meant by ‘reader characteristics’ in the final paragraph of this section.**

Thanks – We have defined a gold-standard for bacteriologically-confirmed TB as:

 

- *“Bacteriologically-confirmed pulmonary tuberculosis, defined as either a documented positive GeneXpert result for Mycobacterium tuberculosison at least one sample of sputum taken for study purposes at Day 56 assessment; or documented growth of Mycobacterium tuberculosisand positive speciation using MPT 64 antigen tests on at least one culture of sputum taken for study purposes at Day 56 assessment; or documented identification of acid fast bacilli on at least one sputum sample taken for study purposes and examined by sputum smear microscopy at Day 56 assessment.”*

 

Then the definition of “not bacteriologically-confirmed” is therefore the inverse of this.

“Reader characteristics” relate to whether radiologists are UK- or Malawi-based, and their years of experience. We have added to the protocol to clarify this.

**11.** **People with previous TB make up a significant proportion of those with TB disease in high burden settings (Florian Marx has done some nice work describing this phenomenon). These people may have residual x-ray changes and there are reports of false positive Xpert tests in individuals who have been recently treated. Will excluding people that have had TB within the preceding six months deal with the latter issue, or can DNA persist for longer than this in people with recent successfully treated TB?**

Thanks – yes, this was the reason for having this exclusion criteria. We agree that it is not perfect, but unfortunately no perfect TB diagnostic currently exists. Through this study, we hope that we can provide evidence for “optimised” (i.e. substantially better than current) diagnostic and treatment linkage approaches.

**12.** **I would like some justification for the Bayesian analysis to be included in the protocol. What is this achieving that couldn’t be achieved using a frequentist approach?**

There is extensive literature describing the potential advantages (and potential complementarity) of Bayesian trial analysis (e.g., for introductory text see: *Bayesian Approaches to Clinical Trials and Health-Care Evaluation *by Speiglehalter, Abrams and Myles, Wiley and Sons, London 2004). 

Some particular advantages of the Bayesian trial analysis include:

 

- Explicit focus on how the trial should change our pre-held conceptions of how effective the trial interventions are (i.e. “a formalisation of learning from experience”). This includes the concept of “credibility of ‘statistically significant’ results once prior belief distributions are incorporated). We think this will be important in aiding decision-makers considering whether interventions should be implemented, and will mitigate against some of the issues raised by the reviewers.
- Given that we are not aware of previous trials of these intervention strategies, we elected to elicit prior beliefs from key stakeholder groups. Prior beliefs of key stakeholder groups important in health decision-making (researchers, policymakers, clinicians, patients, community-members) can be incorporated into analysis, leading to explicit acknowledgement of constraints to acceptance. 
- Posterior distributions intervals from Bayesian analysis are interpretable in a manner than many researchers mistakenly think confidence intervals from frequentist analysis are.

** **


**13. Comments on the trial protocol**

- **It seems like enrollment will precede randomisation but I would like to see a more explicit comment on allocation concealment.**

 

In the protocol, we now state: 

*“* *Enrolment and baseline questionnaires will precede randomization. Randomisation will be done by research assistants using a random number allocation schedule running on study data-collection electronic tablets. Because of the nature of the study and the interventions offered, it will not be possible to blind participants or research assistants to allocation groups. Nevertheless, extensive steps will be taken to ensure that research assistants undertaking outcome assessments are blinded to participants' group. Additionally, the investigators, including the chief investigator and trial statistician, will remain blinded to allocation groups until final analysis. No unblinded interim analysis will be conducted.”*

**14.** **I would like to see more comment about how patients who transfer their care out or are lost to follow up will be handled in the analysis.**

This is described in detail in the Statistical Analysis Plan (Supplemental Material). 

*“Missing data will be examined for each variable and for each individual participant. A systematic assessment of missingness will be conducted to ascertain the reason and possible mechanism for missing data by identifying the quantity of missing data and patterns within the data. Missingness will be compared between randomised arms to assess for systematic biases.*

*Participants who have missing information for outcomes will be excluded from primary analysis. However, in sensitivity analysis, we will use multiple imputation by chained equations to replace missing outcome variables.”*

**15. The CAD threshold that will be used should be pre-specified in the protocol.** 

Thank you. Yes, once the threshold has been established through evaluation of pilot films (to be done with the CAD4TB team in Blantyre in September), we will add this to the protocol.

**16. A note should be made on the approach that will be taken to x-rays in women of childbearing age.** 

In this study, we follow recommendations in the WHO Guidelines for Chest Radiography in Tuberculosis Diagnosis, which state:

*“For pregnant women and the fetus, a CXR does not pose any significant risk, provided that good practices are observed, as the primary beam is targeted away from the pelvis”*

We have recruited experienced radiologists who have received additional training from expert radiologists and x-ray equipment manufacturers, and have procured lead shielding screens to protect the pelvis.

**17. A note should be made about the approach that will be taken to patients that have RpoB mutations detected by Xpert.**

Rifampicin resistance is rare in Blantyre and Malawi and we anticipate detecting very few to no cases. We have added text to state that:

*“Participants with Rifampicin resistance detected on GeneXpert will be traced and supported to access the TB clinic at the Queen Elizabeth Central Hospital for further clinical assessment and evaluation for treatment.”*

**18. It seems unlikely that the trial team will be able to see all participants on day 56. I suggest that the team allow themselves a window of time in which to undertake these study visits.** 

Thanks yes – we have added additional text to make this clear.

**19.** **Please include an explicit comment about whether/how the urine LAM result will be fed back to clinicians.** 

In this section, we refer to undertaking the nested diagnostic accuracy study of a novel manufacturer version of the urine LAM kit (FIND/Fujifilm), which has not yet undergone approval processes. As the performance of this assay is unknown, we will not report these results to clinicians. 

**20. There are a number of grammatical errors and the writing, in places, could be clearer. The section on trial outcomes, in particular, could be better written.** 

Thank you, we have carefully reviewed the manuscript and corrected errors.

****

**Conclusions**

**21.** **The proposed two step approach to evaluating people with suspected pulmonary TB in settings such as these seems to be a good idea. It is good to see this proposal to evaluate the approach in a randomised controlled trial. My main concerns about the protocol concern the choice of primary outcome, particularly how this might perform in a setting where clinicians often initiate empirical TB treatment. An attempt to ascertain ‘true TB status’ would be valuable in interpreting the findings of the trial. The approach that will be taken to define ‘true TB status’ should be described in greater detail. I suggest, as a minimum, that an attempt be made to obtain a baseline sputum sample for TB culture in all those starting TB treatment, regardless of study arm.**

We thank the Reviewer for these helpful suggestions. As discussed above, we don’t aim to define true TB status in all participants in this study, but rather evaluate the cost-effectiveness of interventions that may promote more rapid initiation of TB treatment, and reduce the prevalence of undiagnosed TB and HIV at 8-weeks. Collection of sputum for culture from registering TB cases is not without difficulties, especially where TB treatment will not be initiated by study staff, or may be done at sites other than the study clinic (for example if a participant is admitted to the central hospital, and initiated on TB treatment there). 

We think that the considerable additional cost of additional sputum culture (which is not currently funded), and considerable difficulties in obtaining sputum samples from participants registering for TB treatment under routine conditions, makes this problematic within an already complex study. We suspect that, with these constraints, we would likely have differential ascertainment of culture status at treatment initiation between groups.

# Peer review: Referee response for version 1

**DOI:** 10.21956/wellcomeopenres.15894.r33178

**Published:** 2018-06-04

**Authors:** Frank Cobelens

**Affiliations:** 1 Department of Global Health, Academic Medical Center, Amsterdam, The Netherlands; 2 Amsterdam Institute for Global Health and Development, Amsterdam, The Netherlands

This article describes the rationale, objectives and protocol for a pragmatic, individually randomized 3-arm trial of the effectiveness and cost-effectiveness of optimised HIV and TB diagnosis and linkage to care interventions among adult primary care clinic attendants in Malawi. The trial addresses the question whether HIV self-testing and prompt linkage to HIV care for adults with TB symptoms and a TB triage testing algorithm based on digital chest x-ray with computer-aided diagnosis and Xpert can improve time to treatment initiation. Both are timely and important. In many settings in Africa HIV testing rates remain low, even in patients with presumptive TB, and many patients presenting with cough are not tested for TB despite availability of highly sensitive diagnostics such as Xpert. Triaging based X-ray is a promising approach to increase TB testing levels and case detection, and may have economic benefits over a policy (hardly implemented) of testing all patients meeting the criteria of presumptive TB.

**Is the rationale for, and objectives of, the study clearly described?**

 Yes.

**Is the study design appropriate for the research question?**

 The approach taken in this study is adequate although some concerns can be raised.

**Design:**

- The individually randomized design in a single clinic may introduce a considerable Hawthorne effect, by which routine practice (for HIV and TB testing in arm 1, and for TB testing in arm 2) may much improve over what is expected based on current practice. Although the authors acknowledge this in the discussion, they could have provided more details about how this risk will be mitigated and what the consequences might be in terms of sample size and statistical power.
- Both intervention arms comprise complex interventions in which several elements may be decisive for failure or success. Have authors thought about how these effects could be disentangled? Similarly, it would be interesting to read what safeguards are in place to optimize intervention fidelity.
- What is the currently the proportion of patients started on empirical TB treatment in this setting? As observed in various trials empirical treatment may obscure the effect of introducing Xpert on treatment initiation and outcomes including mortality, and also drive cost-effectiveness estimates. The subgroup analysis stratifying patients into either or not bacteriologically confirmed will shed light on this but the primary endpoint may well be affected.

**Eligibility:** the added value of a triaging approach is mainly in allowing more TB patients to be diagnosed earlier with limited added cost: if more patients reporting at primary care clinics (i.e. with less apparent TB symptoms) would be tested for TB by Xpert this may have major cost or logistics consequences. Triaging would allow identifying patients most at risk for having TB, which will limit the number of Xpert tests that need to be done. However, in order to observe that benefit, eligibility should be sufficiently broad: not only patients who have cough as the presenting symptom, but potentially also those who report a cough regardless of the presenting complaint. It is unclear to what extent this is envisaged. It would also be informative to see how e.g. capacity limitations will be dealt with. If Xpert capacity is e.g. only 20 a day but 40 eligible patients report, how will the 20 be selected? Clearly this should not be based on clinical suspicion of TB.

**Primary endpoint**: time to treatment initiation seems a meaningful patient-important primary endpoint but it does not take into account the numbers of patients started on treatment. If for example in arm 1 three patients are started on TB treatment, all within 2 days, and in arm 300 with a mean of 2 days, the trial will show failure of the intervention. Unless one would count undetected cases at day 56 as started after day 56, but that does not seem the case.

**Adverse events:** one concern that has been raised about the use of CAD is that the algorithms were trained on TB patients, and that other pathology therefore may be missed. Should such occurrences be noted as well?

**Are sufficient details of the methods provided to allow replication by others?**

 Yes. A few details could be described more clearly:

**Eligibility**: see above.

**Cost-effectiveness:** the interpretation of incremental cost-effectiveness ratios (ICERs) for triage strategies is not straightforward. For example the effectiveness in arm 1 or 2 could be higher than in arm 3 if all eligible patients in arm 1 or 2 would have Xpert testing, but with lower cost. It would be useful to see more detail about how ICERs for the triaging interventions will be interpreted.

**Outcome evaluation**: the active follow-up at day 56 is mentioned only late in the article while it is rather vital for understanding the endpoints. I would start with a broad design description that includes this element. Also no mention is made here of standard X-ray at day 56 whereas later paragraphs suggest that it this will be done for the CAD analysis.

**Are the datasets clearly presented in a useable and accessible format?**

 It appears they will be.

**Additional comments **

- The text has a few grammatical errors that could be addressed in a final version.
- The discussion section states that “a triage approach (…) comprises an efficient, high sensitivity initial test, followed by a high specificity confirmatory test”. This does not make sense to me. A test with both high sensitivity and high specificity would be a stand-alone diagnostic. The idea behind triaging is that the test must have high sensitivity but can have moderate specificity (a trade-off with monetary and non-monetary cost).

I have read this submission. I believe that I have an appropriate level of expertise to confirm that it is of an acceptable scientific standard, however I have significant reservations, as outlined above.

# Response

**Published:** 2018-07-23

**Authors:** Peter MacPherson

**Affiliations:** Liverpool School of Tropical Medicine, Malawi

We are grateful for the detailed and helpful reviews provided by Frank Cobelens. We have responded in detail to comments below.

**1.** **The individually randomized design in a single clinic may introduce a considerable Hawthorne effect, by which routine practice (for HIV and TB testing in arm 1, and for TB testing in arm 2) may much improve over what is expected based on current practice. Although the authors acknowledge this in the discussion, they could have provided more details about how this risk will be mitigated and what the consequences might be in terms of sample size and statistical power.**

Thank you for raising this issue. We agree that it is possible that a Hawthorne effect may occur. We carefully considered and ruled out a number of alterative study designs to mitigate against this. One approach would have been to do a clinic cluster-randomised trial; however, with three intervention arms this would be a massive undertaking, and require a substantial additional amount of funding and resources. We additionally considered a before-and-after implementation study, but were concerned that the quality of evidence provided would be substantially lower than that obtained with the current design.

Overall, we believe that the pragmatic study design we selected will provide the highest possible quality evidence to policymakers and researchers with the resources available. If this study demonstrates that interventions are cost-effective, we believe there would a compelling case to evaluate interventions in a larger cluster randomised trial, or as part of evaluation of intervention scale up. On the contrary, if interventions are not effective, this would suggest that further evaluation using these models may not be appropriate.

We have no data to estimate to what extent health workers might modify their practice whilst the study is running, and so are reluctant to speculate about impact on statistical power. However, we have included within the discussion section of the manuscript an additional sentence noting that the possibility of a Hawthorne effect might reduce the size of the differences between the standard of care arm and the interventions arms, and thus power might be reduced. 

Of note, one of the key strengths of the additional Bayesian analysis is that it will allow us to explore the posterior probability of effectiveness of interventions given the (potentially sceptical, neutral, or enthusiastic) prior beliefs elicited from a range of stakeholders (including researchers, clinic staff, and community leaders), allowing us to go some way towards addressing these issues.

**2.** **Both intervention arms comprise complex interventions in which several elements may be decisive for failure or success. Have authors thought about how these effects could be disentangled? Similarly, it would be interesting to read what safeguards are in place to optimize intervention fidelity.**

Our study was carefully designed to ensure that the delivery of interventions would reflect implementation under real-life conditions in low-resource settings. In keeping with the pragmatic trial design, interventions are delivered as a package (e.g. HIV self-testing and subsequent supported linkage to treatment). We believe that it is important that the cost-effectiveness of interventions is evaluated against conditions under which they would be implemented by national HIV and TB programmes in low-resource settings. Although this means that we cannot estimate the effectiveness of any single component of the trial interventions we believe that the evidence provided will be of greater relevance to policymakers grappling with how best to implement new and available HIV and TB diagnostic services. In reality, it would be unattractive if policymakers considered, for example, effectiveness of implementation of only CAD, without considering the additional requirements for confirmatory TB testing and linkage to care. By including combined interventions, we explicitly set out to evaluate the effectiveness and cost-effectiveness of interventions as they would be delivered under real-life conditions. The health economics analysis will allow assessment of the major intervention component drivers of cost-effectiveness.

With regards to ensuring fidelity to interventions: Pragmatic randomised trials explicitly acknowledge that intervention fidelity is not always perfect (e.g. see Sox et al, JAMA 2016). As such they allow estimation of effect sizes that are more realistically representative of implementation under real-life conditions. Nevertheless, as described in the publication and study protocol, we have taken a number of steps to optimise fidelity to interventions. These include: bio-identification of participants (by Simprints fingerprint scanning) at recruitment, prior to randomisation and allocation, prior to delivery of all trial interventions, and at outcome assessment; and exit interviews (when leaving the clinic) conducted on a random sample of 5% of participants to monitor quality of intervention delivery and fidelity to interventions. We will additionally collect data on process indicators, such as the numbers of chest x-rays performed, numbers of HIV tests done, and numbers of sputum samples evaluated.

**3. What is the currently the proportion of patients started on empirical TB treatment in this setting? As observed in various trials empirical treatment may obscure the effect of introducing Xpert on treatment initiation and outcomes including mortality, and also drive cost-effectiveness estimates. The subgroup analysis stratifying patients into either or not bacteriologically confirmed will shed light on this but the primary endpoint may well be affected.** 

We agree that there is likely to be a substantial proportion of patients initiated onto TB treatment empirically, as seen in previous studies. In Blantyre, we estimate that around half of TB patients are started on treatment without microbiological-confirmation. This is likely partly due to epidemiology, but also partly due to limited availability of diagnostics. This situation is common in much of sub-Saharan Africa. We selected the pragmatic trial design specifically acknowledging these facts. By comparing the effectiveness of interventions on time to TB treatment against current standard of care (where rates of empirical treatment are high), we should be able to determine whether optimised HIV testing and TB diagnosis results in more rapid initiation of treatment than what is currently done. Additionally, by testing all participants by TB culture, smear and Xpert at day 56 outcome, we should be able to go some way towards answering the question around appropriateness of TB treatment. Importantly, evidence will be directly relevant to policymakers, as we are comparing effectiveness against the current standard of care in the region TB diagnostic and treatment linkage system.

**4.** **Eligibility:** **the added value of a triaging approach is mainly in allowing more TB patients to be diagnosed earlier with limited added cost: if more patients reporting at primary care clinics (i.e. with less apparent TB symptoms) would be tested for TB by Xpert this may have major cost or logistics consequences. Triaging would allow identifying patients most at risk for having TB, which will limit the number of Xpert tests that need to be done. However, in order to observe that benefit, eligibility should be sufficiently broad: not only patients who have cough as the presenting symptom, but potentially also those who report a cough regardless of the presenting complaint. It is unclear to what extent this is envisaged. It would also be informative to see how e.g. capacity limitations will be dealt with. If Xpert capacity is e.g. only 20 a day but 40 eligible patients report, how will the 20 be selected? Clearly this should not be based on clinical suspicion of TB.**

We agree with these helpful points. Indeed, all acute adult attendees at the study clinic will be screened for the presence of cough, regardless of the reason for attendance – see study screening and eligibility procedures and Figure 1. In terms of capacity, we acknowledge that we may be limited by the availability of onsite GeneXpert testing. In terms of study conduct, we propose that we recruit consecutive daily attendee up to a limit depending upon the capacity of the study team and availability of Xpert, with details to be finalised pending completion of pilot work. We have added text to the paper to describe this. 

*“Where the number of eligible clinic attenders exceeds recruitment capacity, we will recruit participants up to a daily limit, with details finalized pending completion of pilot work showing the number of eligible participants per day.”*

In terms of any potential implementation following the study, we agree that this could be a limiting step. In our health economics analysis, we will model the additional costs and incremental cost effectiveness that would be achieved by National TB Programmes with a broader symptom screening initiative that would subsequently require greater numbers of Xpert tests to be done.

**5. Primary endpoint** **: time to treatment initiation seems a meaningful patient-important primary endpoint but it does not take into account the numbers of patients started on treatment. If for example in arm 1 three patients are started on TB treatment, all within 2 days, and in arm 300 with a mean of 2 days, the trial will show failure of the intervention. Unless one would count undetected cases at day 56 as started after day 56, but that does not seem the case.**

Our previous data (Nliwasa et al, IJTLD 2016) showed that 17% of adults attending the clinic with TB symptoms initiated TB treatment within eight weeks. Since this outcome will be analysis as time-to-event, participants who do not initiate TB treatment will still contribute to analysis, being censored (without experiencing the primary trial outcome) at day 56. As a secondary outcome, we will compare between groups the prevalence of undiagnosed TB (by culture, smear and Xpert) among all participants (regardless of TB treatment status). Although some participants started on TB treatment will have reverted to culture negativity, we hope that this will mitigate against this.

**6.** **Adverse events:** **one concern that has been raised about the use of CAD is that the algorithms were trained on TB patients, and that other pathology therefore may be missed. Should such occurrences be noted as well?**

Thank you – yes. All chest x-rays (in Group 3 at baseline, and in all participants at Day 56) will be clinically reported by Consultant Radiologists, with results reported to patients (by tracing if necessary) and healthworkers where another abnormality is detected.

**7.** **Cost-effectiveness:** **the interpretation of incremental cost-effectiveness ratios (ICERs) for triage strategies is not straightforward. For example the effectiveness in arm 1 or 2 could be higher than in arm 3 if all eligible patients in arm 1 or 2 would have Xpert testing, but with lower cost. It would be useful to see more detail about how ICERs for the triaging interventions will be interpreted.**

We thank the reviewer for raising the issue of how the ICERs for the interventions will be interpreted. We have added to the text to provide more detail of the within-trial cost-effectiveness analysis and how the ICER will be estimated and interpreted. In the example raised by the reviewer where Group 1 or 2 is more effective than Group 3, and associated with lower cost, the interpretation would be that Group 3 would be dominated as it represents a costlier and less effective alternative, and therefore would not be a cost-effective option.

**8.** **Outcome evaluation** **: the active follow-up at day 56 is mentioned only late in the article while it is rather vital for understanding the endpoints. I would start with a broad design description that includes this element. Also no mention is made here of standard X-ray at day 56 whereas later paragraphs suggest that it this will be done for the CAD analysis.**

Thank you. We have added text to the manuscript to give greater prominence to the Day 56 outcome assessment

**8. The text has a few grammatical errors that could be addressed in a final version.**

Thank you, we have carefully reviewed the text and corrected errors.

**10.** **The discussion section states that “a triage approach (…) comprises an efficient, high sensitivity initial test, followed by a high specificity confirmatory test”. This does not make sense to me. A test with both high sensitivity and high specificity would be a stand-alone diagnostic. The idea behind triaging is that the test must have high sensitivity but can have moderate specificity (a trade-off with monetary and non-monetary cost).**

We agree. Here we refer to two separate tests used in sequence in this intervention group: Chest x-ray with CAD, followed by GeneXpert MTB/Rif if CAD indicates that TB is probable.
